# Differential Association Between Surrounding Greenness and Mortality in Individuals With Coronary Heart Disease

**DOI:** 10.1016/j.jacadv.2026.102729

**Published:** 2026-04-09

**Authors:** Gali Cohen, Saar Ashri, Lital Keinan-Boker, Itamar Shafran, Lihi Golan, David M. Broday, David M. Steinberg, Osnat Itzhaki Ben Zadok, Tamir Bental, Mika Moran, Ran Kornowski, Yariv Gerber

**Affiliations:** a Department of Epidemiology and Preventive Medicine, School of Public Health, Gray Faculty of Medical and Health Sciences, Tel Aviv University, Tel Aviv, Israel; b Israel Center for Disease Control, Israel Ministry of Health, Israel; c School of Public Health, University of Haifa, Israel; d Technion Center of Excellence in Exposure Science and Environmental Health, Technion Israel Institute of Technology, Israel; e Department of Statistics and Operations Research, Tel Aviv University, Tel Aviv, Israel; f Department of Cardiology, Rabin Medical Center (Beilinson and Hasharon Hospitals), Israel

**Keywords:** coronary heart disease, epidemiology, greenness, mortality, NDVI, walkability

## Abstract

**Background:**

Residential greenness has been linked to lower mortality; however, whether this association varies by cardiovascular disease status remains unclear.

**Objectives:**

The objectives of the study were to examine the association between greenness and mortality among individuals with and without coronary heart disease (CHD), distinguishing between stable and acute patients, and to assess modification by individual- and neighborhood-level factors, including walkability.

**Methods:**

We analyzed data from 4 Israeli cohorts: 2 CHD patient-based cohorts and 2 population-based cohorts (inception years 1992-2004; mortality follow-up through 2020). Long-term greenness exposure was estimated using satellite-based Normalized Difference Vegetation Index within an 800m buffer around participants’ homes.

**Results:**

We included 17,431 participants (mean [SD] age 63.6 [15.3] years; 5,286/17,431 [30.3%] women), of whom 3,959/17,431 (22.7%) were CHD-free, 4,771/17,431 (27.3%) had stable CHD, and 8,701/17,431 (49.9%) had acute CHD at baseline. During a median (IQR) follow-up of 11.8 (7.6-15.7) years, 6,055 deaths occurred. Greenness was associated with lower mortality among stable CHD (HR: 0.90; 95% CI: 0.81 to 0.99, per IQR increase in normalized difference vegetation index) and acute CHD (HR: 0.88; 95% CI: 0.82-0.94), but not among CHD-free individuals (HR: 1.01; 95% CI: 0.88-1.16) (*P*_interaction_ = 0.004). Stratified analysis among all participants demonstrated strongest greenness-mortality association in the high walkability tertile (HR: 0.83; 95% CI: 0.75-0.91) compared with the medium (0.95; 0.88-1.04) and low (0.90; 0.82-0.98) tertiles (*P*_interaction_ = 0.05).

**Conclusions:**

Residential greenness is associated with better survival among individuals with CHD. Variation by walkability suggests that built environment characteristics may modify greenness-related health benefits.

## Background

The susceptibility of individuals with pre-existing coronary heart disease (CHD) to adverse urban exposures is well established.^1^, ^2^, ^3^ Both the onset and progression of CHD have been associated with air pollution,^4^, ^5^, ^6^ heat stress,^7^^,^^8^ and noise,^9^^,^^10^ along with abundant evidence linking air pollution to increased mortality among individuals with various cardiovascular conditions, including myocardial infarction (MI),^6^^,^^11^^,^^12^ acute coronary syndrome,^13^ heart failure (HF),^14^ and following cardiac transplantation.^15^ Evidence further indicates that the association between air pollution and mortality differs between individuals with and without CHD, with consistently enhanced mortality risk observed among those with pre-existing CHD for both short-^16^ and long-term^17^ air pollution exposure. Given this increased vulnerability to harmful environmental exposures, individuals with CHD may also derive greater benefits from health-promoting environments, such as natural green spaces.

Green spaces play a crucial role in shaping health outcomes, particularly in urban settings where exposure to harmful environmental stressors is unavoidable.^18^ A recent meta-analysis comprising more than 8 million individuals from different countries has demonstrated a significant inverse relationship between surrounding greenness and the risk of all-cause mortality.^19^ Extending this evidence to cardiovascular outcomes, a large-scale meta-analysis spanning 18 countries and more than 100 million individuals reported that higher levels of greenness were associated with lower odds of cardiovascular outcomes, including cardiovascular mortality, ischemic heart disease mortality, cerebrovascular disease mortality, and stroke incidence or prevalence.^20^ This protective association is partly attributed to the ability of green spaces to mitigate harmful urban exposures, including air pollution,^21^ noise,^22^ and urban heat island effects,^23^ while also fostering physical activity and social cohesion,^24^ both of which are essential for maintaining cardiovascular health.^25^^,^^26^ Beyond these indirect pathways, visual exposure to green environments has been directly associated with improved recovery and hospitalization outcomes in diverse patient populations,^27^, ^28^, ^29^ suggesting a stress-reduction mechanism that may be particularly relevant for individuals with CHD.^30^ Given this dual role—reducing harmful exposures and promoting physiological and psychosocial resilience through stress reduction, physical activity, and social cohesion—green spaces may provide substantial health benefits, especially for those with pre-existing CHD.^31^

Cohort studies investigating the association between green spaces and mortality among CHD populations have yielded mixed results, with some indicating no association,^32^ and others reporting protective associations across different CHD subgroups and settings.^33^, ^34^, ^35^ In this study, we examined the association between greenness and mortality among individuals with and without CHD, distinguishing between stable and acute conditions. We further explored whether individual and neighborhood-level characteristics modify this association.

## Methods

### Study population

We obtained data from 4 different cohorts: 2 patient-based cohorts with CHD and 2 population-based cohorts. The data collection and integration process has been previously described.^17^ One CHD cohort comprises all individuals (n = 1,521) aged ≤65 years who were hospitalized for incident MI between 1992 and 1993 in 1 of the 8 hospitals in central Israel.^36^ The other CHD cohort is a patient-based administrative database comprising all consecutive patients who underwent percutaneous coronary interventions (PCIs) at the cardiology department of Rabin Medical Center in Israel between 2004 and 2014 (n = 12,784).^37^ The 2 additional cohorts are national samples from the Israeli National Health and Nutrition Surveys, conducted by the Israel Ministry of Health, with participants aged 25 to 64 (n = 3,246; inception years: 1999-2001)^38^ and aged 65+ (n = 1,799; 2005-2006).^39^ Survey participants who reported any pre-existing cardiovascular condition at baseline (including prior MI/chronic HF/other CHD/bypass surgery/PCI) or those without information on CHD status were excluded (n = 804). In all cohorts, participants with missing information on greenness exposure or mortality were excluded from the analysis. The appropriate institutional ethics committees approved all aspects of the study.

### Classification of CHD status

We classified all participants into 3 groups according to pre-existing CHD status: CHD-free, stable CHD, and acute CHD. Following the exclusion of patients with self-reported baseline CHD, all the participants in the population-based cohorts were classified as CHD-free. Patients in the MI cohort were all classified as having acute CHD. Within the PCI cohort, patients undergoing urgent PCI for acute/recent MI or unstable angina pectoris were classified with acute CHD. In contrast, those undergoing an elective PCI were classified as stable CHD.

### Mortality ascertainment

Mortality data (last updated in December 2020) were obtained for all 4 cohorts by linking participants’ national identification numbers to the National Mortality Database, managed by the Israeli Ministry of Health and accessed through TIMNA, Israel’s national big data platform for health research. TIMNA includes anonymized health information derived from national registries and enables linkage using national identification numbers without disclosing any personally identifying information to researchers.

### Greenness exposure

We estimated residential greenness using the normalized difference vegetation index (NDVI). NDVI is a remotely sensed spectral index derived from satellite imagery that quantifies vegetation cover over a unit area. Chlorophyll absorbs red light (R) (∼0.66 μm), whereas healthy vegetation reflects near-infrared light (NIR) (0.7-1.1 μm). NDVI, calculated as: (NIR − R)/(NIR + R), captures this contrast, ranging from −1.0 to 1.0, with higher values indicating greater vegetative density.^40^ We obtained imagery data from Landsat 5 L2 C2 and Landsat 8 L2 C2 satellites (downloaded from the United States Geological Survey EarthExplorer website at 30m × 30m spatial resolution^41^) for each available year between 1992 and 2019. To capture peak greenness, we used spring (March-May) images^42^ with <10% cloud cover, ensuring maximum vegetation visibility.^43^ To achieve complete coverage of the study area (north, center, and south), we combined 3 images per available year (Supplemental Table 1) and calculated annual NDVI for the entire country using ArcGIS Pro (Supplemental Figure 1). Because interannual NDVI estimates at the same residence were highly correlated and short-term variability may reflect measurement or phenological fluctuations, we defined long-term exposure as the average NDVI over the follow-up period to better characterize chronic residential greenness. To capture greenness exposure in the neighborhood, we used the mean NDVI within an 800 m circular buffer around each residence. The optimal buffer size is uncertain, particularly given our diverse study population with varying CHD status, mobility patterns, and greenness-related benefits.^24^ We chose the 800 m buffer for primary analysis as it likely reflects broader environmental characteristics relevant to all groups. To test the robustness of results, we additionally calculated the mean NDVI for smaller buffer zones (300 m and 100 m).

### Walkability Measures

To account for built environment characteristics, we calculated a walkability index that reflects the level of neighborhood support for walking as a mode of transportation and recreation.^44^ The index incorporated 3 components^45^ within an 800 m buffer around each participant’s home: 1) population density, indicating the number of residents per unit area; 2) street connectivity, measured as the number of intersections, reflecting ease of pedestrian movement; and 3) land-use mix, assessed using the entropy index to capture destination diversity (Supplemental Figure 2).^46^ We obtained data from the Israel Central Bureau of Statistics and Open Street Map via Geographic Information System (ArcGIS Pro 2.8.0). To create a composite walkability index, we standardized these components, assigning greater weight to street connectivity because of its pivotal role in facilitating pedestrian movement and enhancing walkability.^44^^,^^45^ We then summed them to derive the final index.^47^ A detailed description of the walkability index calculation is presented in the Supplementary Materials.

### Traffic-Related air Pollution

We estimated long-term traffic-related air pollution using a national high-resolution land-use regression model (50 × 50 m resolution), providing annual mean nitrogen oxides (NO_x_) levels (cross-validated R^2^ = 0.74).^48^ Given high correlations between annual estimates (*r*_p_ > 0.85) and residual correction availability from 2000 onward,^49^ we assigned participants' mean annual NO_x_ concentrations at their residence for 2000 to 2012.

### Other covariates

Demographic and clinical data were collected at baseline through interviews in the population cohorts, via electronic medical records in the PCI cohort, and through both methods in the MI cohort. Covariates included in the current study were selected based on their availability across all cohorts and then harmonized across cohorts. Baseline covariates included age, sex, ethnicity (Arab, Jew, or other), smoking (self-reported status of smoker/nonsmoker), and chronic morbidities including diabetes mellitus, hypertension, and previous stroke, assessed via self-report in MABAT cohorts and via electronic medical records in the patient-based cohorts. Neighborhood socioeconomic status (SES) was estimated through a 20-point scale developed by the Israel Central Bureau of Statistics, based on the 1995 national census, with a score of 20 representing the highest neighborhood SES.^50^ Urbanity was defined as a dichotomous indicator based on Central Bureau of Statistics criteria, classifying localities with over 20,000 inhabitants as an urban municipality.^51^

### Statistical analysis

We compared baseline characteristics between included and excluded participants, across cohorts, and by NDVI tertiles. Differences between groups were assessed using t-tests or one-way analysis of variance for continuous variables and chi-square tests for categorical variables. Standardized mean differences (were used to enable interpretable comparison of group differences across variables.^52^ To assess missing data patterns, we first quantified the extent of missingness across study variables. Missing data were concentrated in environmental variables (ie, neighborhood SES [7.5%], annual NO_x_ levels [5.6%], and the walkability index [8.4%]), whereas missingness in clinical characteristics was minimal (<1%). On this basis, we compared baseline characteristics between participants with and without available data for these environmental variables, stratified by CHD category. The Pearson correlation coefficient was used to assess the correlation between annual exposure estimates and spatial scales. Survival probabilities by NDVI tertiles were visualized using Kaplan-Meier curves within each CHD category, and differences between curves were assessed using the log-rank test. Cox proportional hazards regression models estimated HRs and 95% CIs for all-cause mortality per an interquartile (IQR) increase in NDVI, using age as the time scale.^53^ We chose age over time-on-study to standardize follow-up, given different cohort entry points (hospital admission vs interview). We ran 3 models in each CHD category: 1) minimally adjusted models, accounted for age (via time scale) and sex; 2) multivariable-adjusted models, additionally adjusted for ethnicity, neighborhood SES, smoking, diabetes, hypertension, stroke, and year of study entry; and 3) fully adjusted models, further adjusted for urbanity, NO_x_ levels, and walkability index. We examined effect modification by testing a two-way interaction term of NDVI by CHD in a multivariable-adjusted model, which also included NDVI and CHD status as main effects in the overall study population. We also ran models using a categorical exposure classification based on NDVI tertiles, with the lowest tertile serving as the reference category. In models that included participants from multiple cohorts, we fitted stratified Cox proportional hazards models with cohort-specific baseline hazards to account for potential between-cohort heterogeneity.^54^ Proportional hazards assumptions were tested using Schoenfeld residuals, and no violations were detected. To examine possible nonlinearity in the greenness-mortality association, we modeled NDVI as a penalized spline with 3 degrees of freedom and evaluated the model fit using partial log-likelihood tests. These analyses were performed across all levels of model adjustment. We evaluated evidence for nonlinearity using a likelihood ratio test comparing models with NDVI modeled as a penalized spline vs a linear NDVI term.

To examine effect modification by individual characteristics (age and sex) and contextual factors (neighborhood SES and walkability), we assessed the greenness-mortality association across strata within the entire cohort and within each CHD status group separately. *P* values for the interaction were derived using likelihood ratio tests, comparing the full model with the NDVI∗modifier interaction term to a nested model without it. The test statistic was evaluated using a chi-square distribution with degrees of freedom equal to the number of interaction terms added.^55^

We conducted several sensitivity analyses to assess the robustness of our main findings. First, we evaluated the impact of a different exposure window, defined as NDVI levels during the first 2 years of follow-up. This window was selected because it may be particularly relevant in the context of CHD populations, given stress-reduction mechanisms associated with exposure to natural environments that may improve recovery and hospitalization outcomes.^35^ Since CHD-free individuals did not have a comparable clinical index event at baseline, we assessed NDVI during the first 2 years following the interview for comparability. Second, we calculated a trimmed mean, excluding the 2 lowest and the 2 highest values from the long-term average, to provide a more stable estimate of long-term exposure. Third, given the uncertainty regarding the relevant exposure spatial scale,^24^ we repeated the analyses using 300- and 100-m buffers, adjusting for walkability at the corresponding spatial scale (400-m buffer). Finally, to assess potential differences within the acute CHD group, we conducted separate analyses for patients hospitalized due to MI and unstable angina pectoris. Analyses were performed using R software (R Foundation for Statistical Computing).

## Results

Analysis included 17,431 participants (mean [SD] age at baseline, 63.6 [15.3]; 5,286/17,431 [30.3%] women). No substantial differences were observed between included and excluded participants, except for lower mean (SD) NO_x_ levels among excluded compared with included participants (22.0 [11.4] vs 25.4 [9.7] ppb) (Supplemental Table 2). Of the included participants, at baseline, 3,959/17,431 (22.7%) reported no CHD, 4,771/17,431 (27.4%) were hospitalized for PCI with no acute indication (therefore categorized as stable CHD), and 8,701/17,431 (49.9%) were hospitalized with acute CHD. The median (IQR) follow-up time for all participants was 11.8 (7.6-15.7) years, during which 6,055 deaths occurred. Of those, 820/3,959 (20.7%) occurred among individuals free of CHD at baseline, 1,824/4,771 (38.2%) among those with stable CHD, and 3,411/8,701 (39.2%) among acute patients.

Individual characteristics substantially differed across CHD groups (Supplemental Table 3): CHD patients were older (mean [SD] age: 69.6 [11.3] in stable and 65.7 [13.0] in acute vs 51.4 [17.5] in CHD-free) and less likely to be female (23.2% in stable and 23.3% in acute vs 54.3% in CHD-free). CHD patients also had substantially higher prevalence of CVD-related risk factors and comorbidities, including smoking, diabetes, hypertension, and stroke. Similar patterns were observed at the cohort level (Supplemental Table 4). The proportion of Arab participants was lower in CHD patient-based cohorts than in population-based cohorts, reflecting intentional oversampling in the national surveys. Environmental exposures also varied across cohorts, with the most pronounced differences observed for residential NO_x_ concentrations, which were highest in the MI cohort. Comparison between participants with and without data on environmental covariates (neighborhood SES, NO_x_, and walkability data) showed differences in contextual but not clinical characteristics, specifically higher levels of urban-related exposures (Supplemental Tables 5 to 7).

The distribution of characteristics across exposure tertiles is presented in Table 1. Participants in the lowest NDVI tertile were more likely to live in lower-SES neighborhoods (mean [SD] score 10.9 [3.6] in the lowest vs 11.7 [4.2] and 11.9 [4.2] in the medium and high tertiles). They were also slightly more likely to be female (1,871/5,811 [32.2%] vs 1,796/5,811 [30.9%] and 1,619/5,809 [27.9%]) and smokers (2,040/5,811 [35.1%] vs 1,925/5,811 [33.2%] and 1,943/5,809 [33.5%]). This pattern was consistent across CHD categories (Supplemental Table 8). The mean (range) NDVI within an 800m buffer was 0.125 (0.016-0.279). The distribution of characteristics across exposure tertiles is presented in Table 1.

**Table 1:** Pooled Cohort Characteristics by Greenness Exposure Tertile

NDVI Tertiles	Low (n = 5,811)	Medium (n = 5,811)	High (n = 5,809)	SMD
Mean (range) NDVI	0.089 (0.016, 0.107)	0.122 (0.107, 0.137)	0.165 (0.137, 0.279)	
Age, y, mean ± SD	64.5 (15.6)	63.1 (15.2)	63.0 (15.1)	0.06
Female, n (%)	1,871 (32.2)	1,796 (30.9)	1,619 (27.9)	0.06
Neighborhood SES, mean ± SD	10.9 (3.6)	11.7 (4.2)	11.9 (4.2)	0.16
Arab ethnicity, n (%)	676 (11.6)	673 (11.6)	472 (8.1)	0.08
Smoking, n (%)	2,040 (35.1)	1,925 (33.2)	1,943 (33.5)	0.03
Diabetes, n (%)	2,043 (35.2)	1,916 (33.0)	2,043 (35.2)	0.03
Hypertension, n (%)	3,542 (61.2)	3,374 (58.5)	3,428 (59.4)	0.04
Stroke, n (%)	318 (5.5)	260 (4.5)	240 (4.1)	0.04
Nitrogen oxides, ppb, mean ± SD	29.3 (9.7)	25.6 (9.2)	21.1 (8.3)	0.61
Walkability level, n (%)				0.45
Low	1,144 (20.7)	1,790 (32.6)	2,384 (48.0)	
Medium	1,843 (33.4)	1,889 (34.4)	1,600 (32.2)	
High	2,530 (45.9)	1,807 (32.9)	981 (19.8)	
Living in urban locality, n (%)	5,735 (98.8)	5,560 (96.5)	3,812 (68.8)	0.61

All comparisons across NDVI tertiles were statistically significant (*P* < 0.05), except for smoking (*P* = 0.06); however, SMDs are reported to enable interpretable comparison of group differences across variables. Reported SMDs represent the average of all pairwise comparisons across NDVI tertiles.

NDVI = normalized difference vegetation index; ppb = parts per billion; SES = socioeconomic status; SMD = standard mean difference.

NDVI values across spatial scales (100m, 300m, and 800m buffers) were highly correlated, with Pearson r coefficients ranging between 0.81 and 0.93. Annual NDVI estimates further showed strong intercorrelations, with a median (25th–75th percentile) Pearson r coefficient of 0.86 (0.77-0.92) (Supplemental Figure 3). Most participants (15,107/17,431 [86.7%]) lived in urban areas, and the mean (range) of the walkability index (standardized composite score) in an 800m buffer was 0.1 (−7.1 to 16.1).

**Figure 1:**
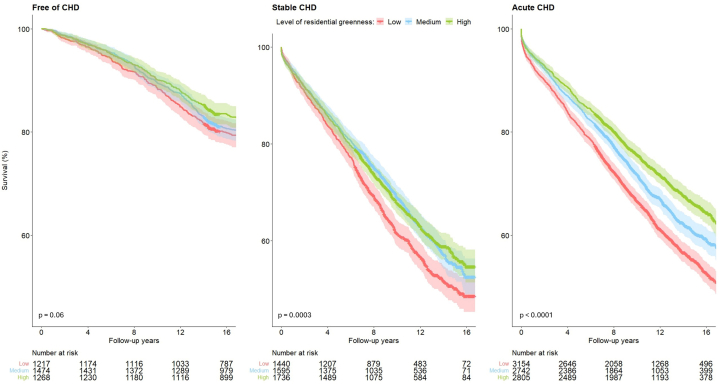
**Survival by Residential Greenness Across Coronary Heart Disease Status** Survival (%) according to the level of residential greenness (indicated by 800m-NDVI tertiles) among individuals free of CHD, stable CHD and acute CHD patients. CHD = coronary heart disease.

Survival rates increased across NDVI tertiles, with the greatest differences observed in patients with acute CHD (Figure 1 and Central Illustration). Among CHD-free individuals, 15-year survival estimates were 0.80 (95% CI: 0.78-0.83) in the lowest tertile, 0.82 (95% CI: 0.79-0.84) in the medium, and 0.84 (95% CI: 0.82-0.86) in the highest (*P* = 0.06). Corresponding survival estimates in stable CHD were 0.50 (95% CI: 0.47-0.53), 0.55 (95% CI: 0.52-0.58), and 0.57 (95% CI: 0.54-0.60) (*P* < 0.001), and in acute CHD, 0.55 (95% CI: 0.53-0.57), 0.60 (95% CI: 0.58-0.63), and 0.66 (95% CI: 0.64-0.68) (*P* < 0.001).

**Central Illustration:**
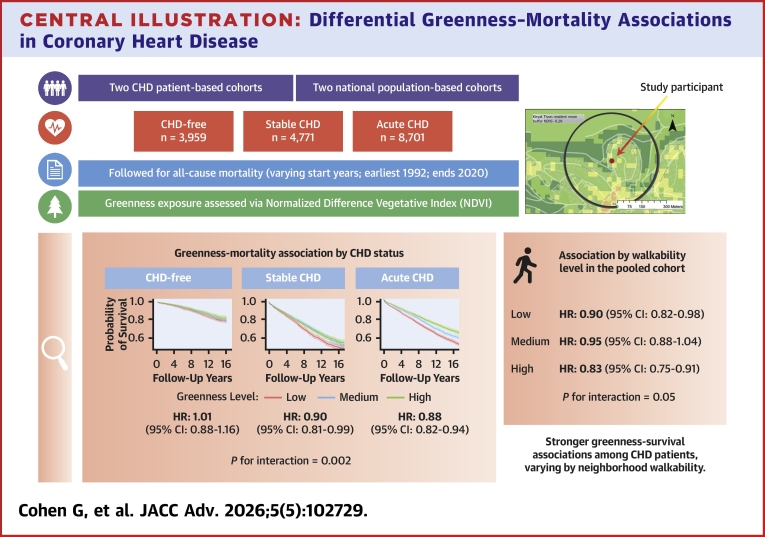
**Differential Greenness–Mortality Associations in Coronary Heart Disease** Central illustration summarizing a multicohort analysis of residential greenness and all-cause mortality among 17,431 individuals with and without coronary heart disease (CHD) from 4 Israeli cohorts (inception years 1992-2004; mortality follow-up through 2020). Residential greenness was assessed using satellite-derived normalized difference vegetation index (NDVI) around participants’ homes. Associations were examined separately among CHD-free individuals and patients with stable or acute CHD. Higher greenness was associated with lower mortality among individuals with stable and acute CHD, but not among those without CHD. Neighborhood walkability emerged as a modifier, with stronger protective associations observed in highly walkable environments. Findings highlight differential vulnerability and benefit across clinical subgroups and underscore the combined influence of natural and built environments on survival among patients with CHD.

**Figure 2:**
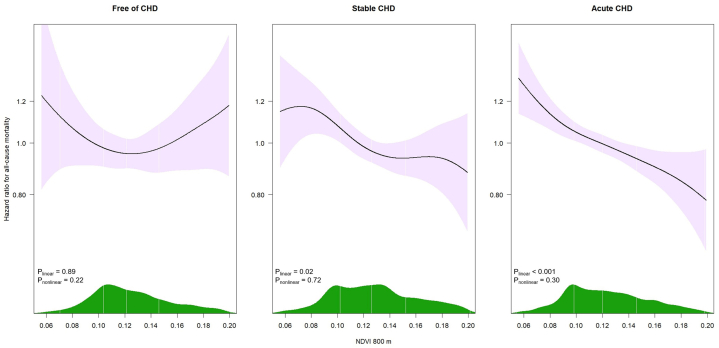
**NDVI–Mortality Exposure–Response Curves by Coronary Heart Disease Subgroup** Exposure-response curves for the association between 800 m NDVI and all-cause mortality in each CHD subgroup. Curves are based on Cox models with age as the time scale including a penalized spline term for NDVI (3 degrees of freedom). Models were adjusted for sex, smoking, pre-existing comorbidities (diabetes, hypertension, and stroke), neighborhood SES, year of study entry, living in an urban locality, traffic-related air pollution, and walkability index. The histograms at the bottom show NDVI exposure distribution in each CHD category. The lowest and highest 2.5% exposure percentiles were trimmed for visualization. Solid lines represent HR estimates and shaded areas indicate 95% CIs. P_linear_ denotes the *P* value for a linear association between NDVI and mortality and P_nonlinear_ the *P* value for deviation from linearity, both obtained from likelihood ratio tests. Abbreviation as in Figure 1.

Associations between residential greenness and mortality varied by CHD group (Table 2 and Central Illustration). Among CHD-free individuals, a 1-IQR increase in NDVI (0.047) was associated with a modest, nonsignificant reduction in mortality risk in the minimally adjusted model, but the association attenuated to the null in the fully adjusted model. In the stable CHD group, associations were null in the minimally and partially adjusted models but became protective in the fully adjusted model (HR: 0.90; 95% CI: 0.81-0.99). Among acute CHD patients, protective associations were consistently observed across all degrees of adjustment (HR: 0.88; 95% CI: 0.82-0.94 in the fully adjusted model). A statistically significant interaction between greenness and CHD status was observed (*P*_interaction_ = 0.002). When modeling NDVI as tertiles (with the lowest as the reference), we observed no clear association with mortality among CHD-free individuals, whereas a consistent association was found among stable patients in both the medium and upper NDVI tertiles. Among acute patients, the association attenuated to the null in the middle NDVI tertile after full adjustment, but remained robust in the upper tertile (HR: 0.83; 95% CI: 0.75-0.92). Spline analyses indicated no significant nonlinear components in any CHD subgroup (*P*_CHD-free_ = 0.22; *P*_Stable_ = 0.72; *P*_Acute_ = 0.30). The concentration-response curves for the fully adjusted model (Figure 2) showed that among CHD-free individuals, an inverse association between greenness and mortality was evident from the minimum NDVI value up to the 50th percentile (0.122), with the curve changing direction at higher values, with wide CIs. In stable patients, a protective association was observed primarily between the 10th and 70th percentiles of the NDVI (0.087-0.141), whereas among acute CHD patients, the association appeared protective and nearly linear across the entire exposure distribution. Similar patterns were observed across all levels of model adjustment (data not shown).

**Table 2:** Association Between Greenness Exposure (Indicated by Normalized Difference Vegetation Index in 800 m Around Residential Location) and All-Cause Mortality by Coronary Heart Disease Status (N = 17,431)

	Free of CHD (n = 3,959, 820 Events)			Stable CHD (n = 4,771, 1,824 Events)			Acute CHD (n = 8,701, 3,411 Events)		
	Model 1^a^	Model 2^b^	Model 3^c^	Model 1^a^	Model 2^b^	Model 3^c^	Model 1^a^	Model 2^b^	Model 3^c^
Greenness modeled on a continuous scale									
-	0.92 (0.83-1.02)	1.02 (0.91-1.14)	1.01 (0.88-1.16)	0.99 (0.94-1.05)	0.99 (0.92-1.05)	0.90 (0.81-0.99)	0.86 (0.82-0.90)	0.87 (0.83-0.92)	0.88 (0.82-0.94)
Categorical classification of greenness exposure									
Low	1.00	1.00	1.00	1.00	1.00	1.00	1.00	1.00	1.00
Medium	0.84 (0.71-0.98)	0.94 (0.80-1.11)	0.94 (0.79-1.13)	0.91 (0.81-1.02)	0.90 (0.80-1.01)	0.87 (0.77-0.99)	0.89 (0.82-0.96)	0.93 (0.86-1.01)	0.96 (0.88-1.05)
High	0.83 (0.70-0.99)	1.00 (0.84-1.21)	1.00 (0.81-1.24)	0.92 (0.82-1.03)	0.92 (0.82-1.03)	0.84 (0.72-0.97)	0.78 (0.71-0.84)	0.81 (0.74-0.88)	0.83 (0.75-0.92)
*P*_for interaction_ = 0.002^d^									

Measured in a continuous scale, greenness scaled to a 1–IQR increment (0.047). Sample size and the number of events are reported for the full analytic dataset.

CHD = coronary heart disease.

a Adjusted for sex (age was accounted for in the time scale).

b Additionally adjusted for ethnicity, smoking, preexisting comorbidities (diabetes, hypertension and stroke), neighborhood SES and year of study entry.

c Additionally adjusted for living in an urban locality, exposure to traffic-related air pollution, and walkability index measured within a buffer of 800m around residential location [excluding 266 (6.7%) free of CHD, 828 (17.4%) stable and 1,403 (16.1%) acute participants due to missing information on these variables).

d P for interaction was derived from a multivariable-adjusted model including an NDVI-by-CHD interaction term in the overall study population.

**Figure 3:**
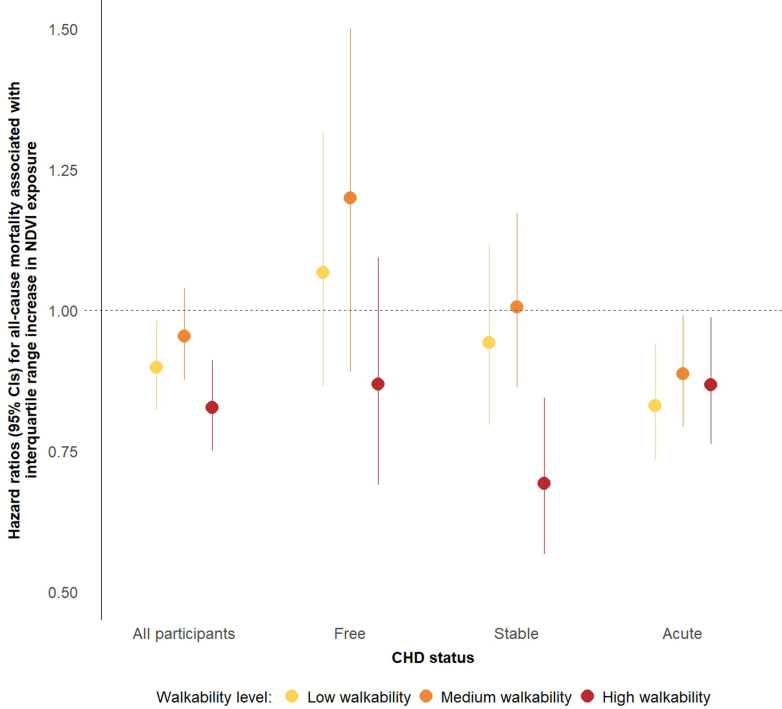
**Greenness–Mortality Association by Walkability and Coronary Heart Disease Status** Association between greenness exposure, as indicated by NDVI in an 800 m radius around residential locations, and all-cause mortality, stratified by walkability tertile, among all participants, and by CHD status. HRs are presented for a 1-IQR increase in NDVI (0.047). Models used age as the time scale and were adjusted for sex, smoking, pre-existing comorbidities (diabetes, hypertension, and stroke), neighborhood SES, year of study entry, living in an urban locality, and exposure to traffic-related air pollution. *P* values for interaction were derived using likelihood ratio tests, comparing the full model with the NDVI∗walkability interaction term to a nested model without it. *P* values for interaction: All participants, *P* = 0.05; CHD-free, *P* = 0.03; stable CHD, *P* = 0.16; acute CHD, *P* > 0.20. NDVI, normalized difference vegetation index; other abbreviation as in Figure 1.

In a stratified analysis by walkability (Figure 3), the magnitude of the greenness-mortality association among all participants varied by walkability level, with the strongest association seen in the highest walkability tertile (HR: 0.83; 95% CI: 0.75-0.91), compared with the lowest (HR: 0.90; 95% CI: 0.82-0.98) and medium (HR: 0.95; 95% CI: 0.88-1.04) tertiles (*P*_interaction_ = 0.05). Within CHD subgroups, the greatest differences across walkability tertiles were observed among stable CHD patients, with the association substantially stronger in the highest walkability tertile (HR: 0.69; 95% CI: 0.57-0.84) and attenuated toward the null in lower tertiles (*P*_interaction_ = 0.16). Among CHD-free individuals, there was a protective association in the high-walkability category, yet with CI including unity (HR: 0.87; 95% CI: 0.69-1.09; *P*_interaction_ = 0.03). The protective greenness-mortality association among acute CHD patients was similar across walkability categories, with HR: 0.83 (95% CI: 0.73-0.94), HR: 0.89 (95% CI: 0.79-0.99), and HR: 0.87 (95% CI: 0.76-0.99) in the lowest, medium, and highest walkability tertiles, respectively (*P*_interaction_ > 0.20). Stratified analyses by age, sex, and neighborhood SES did not reveal substantial differences across strata, either in the full sample or within CHD subgroups (Supplemental Figures 3 to 5). Notably, there was some indication for a slightly stronger association among individuals under 65, particularly among the stable patients, and in both the lowest and highest neighborhood SES tertiles in the overall sample.

### Sensitivity analysis

Using NDVI at the first 2 years of follow-up, we observed no substantial differences from the main analysis for CHD-free and stable CHD groups, whereas among acute patients, the association between NDVI and mortality was attenuated (fully adjusted HR: 0.94; 95% CI: 0.89-1.00) (Supplemental Table 9). Assessing exposure using the trimmed mean approach yielded consistent results with the main analysis across all CHD groups (Supplemental Table 9). Repeating the primary analysis with NDVI calculated at alternative spatial scales produced similar findings for the 300m buffer, whereas the association among CHD patients was slightly attenuated for the 100 m buffer (HR _Stable_: 0.93; 95% CI: 0.85-1.02; HR _Acute_: 0.91; 95% CI: 0.86-0.97) (Supplemental Table 10). Analyzing MI and unstable angina pectoris separately showed no major differences between groups (Supplemental Table 11).

## Discussion

In this study, we examined whether the association between residential greenness and mortality risk varies by CHD status. Using a large, pooled cohort data set, we found an association between increased greenness and improved survival among individuals with CHD. Among acute CHD patients, this association persisted across all levels of adjustment, whereas in stable CHD patients, the association became stronger after adjusting for individual- and neighborhood-level characteristics. Among CHD-free individuals, an initial protective association in minimally adjusted models was no longer observed after multivariable adjustment. The previous findings remained robust across multiple sensitivity analyses. Stratified analyses by walkability revealed a stronger association between greenness and mortality among individuals living in high-walkability areas.

Our results of an enhanced protective association among individuals with CHD are consistent with the well-established susceptibility of CHD patients to environmental exposures.^1^ However, a previous study conducted in Ontario, Canada,^32^ also comparing CHD and CHD-free individuals, obtained different results. It found no association between greenness and cardiovascular outcomes (including hospital readmission for any cardiovascular cause and cardiovascular mortality) among individuals with pre-existing MI or HF. In contrast, an inverse association was observed between greenness and cardiovascular outcomes (including the incidence of MI and HF, as well as cardiovascular mortality) among individuals who were free from these conditions. Notably, effect modification by CHD status was not explicitly tested. This null association among CHD individuals contrasts not only with our results, but also with previous cohort-based findings that showed a protective association of greenness on mortality in varying CHD populations: MI and stroke survivors in Northern California,^34^ stroke survivors in the Greater Boston area,^33^ and patients who underwent coronary artery bypass graft surgery in Israel.^35^ Beyond interstudy variability (eg, different cohort characteristics, exposure assignment, and confounding adjustment), local climate conditions may also explain the observed differences between studies—particularly temperature-related patterns in park utilization.^56^ Given that individuals with pre-existing CHD experience higher cardiac workload during exercise in the cold,^57^ it is possible that CHD patients in colder areas as in the study from Canada^32^ may be more likely to avoid outdoor exercise compared with CHD individuals living in warmer climates such as in our study and previous studies from Northern California^34^ and Israel,^35^ where outdoor green environments are accessible nearly year-round. Moreover, in hot climates, the crucial role of vegetation in reducing heat load may further explain our results. Urban parks, particularly those with high trees and broad canopies, were shown to reduce temperatures in the Tel Aviv area by up to 3.5 °C.^58^ As CHD patients are particularly vulnerable to heat,^59^ the shade and cooling effects of vegetation may be especially important in promoting physical activity or providing general health benefits for this population.^60^

Our results for the CHD-free group indicated an inverse association in base models, which became null after adjustment for individual and environmental factors. Although a large body of evidence suggests a protective association of greenness with mortality among the general population,^19^^,^^32^^,^^61^ our results are consistent with studies conducted in Australia^62^ and Italy^43^ that reported close-to-null associations. Variations in NDVI exposure assignment and confounder adjustment strategies may account for these divergent findings. In our study, we used a single NDVI value for each year in a follow-up period exceeding 20 years. In contrast, some previous studies relied on shorter follow-up periods or a single-year NDVI image for the entire follow-up.^43^^,^^61^ Furthermore, our models were adjusted for various factors that were not consistently accounted for in previous investigations, including preexisting comorbidities, neighborhood SES, air pollution, and walkability index.

Neighborhood walkability emerged as a modifier of the greenness-mortality association, with a stronger protective association among individuals who lived in highly walkable neighborhoods. These findings underscore the critical role of the built environment characteristics in modulating greenness accessibility. In high-walkable areas, the walking-friendly infrastructure may facilitate the use of nearby green spaces for outdoor activities. However, in low-walkable neighborhoods, even when parks are close, poor accessibility may limit their use and associated benefits. This pattern was evident in the overall sample and driven primarily by individuals with stable CHD, whereas among acute patients the greenness-mortality association was observed across all walkability levels. Factors related to acute clinical conditions may influence the extent to which individuals depend on pedestrian-friendly, supportive neighborhood environments. Among nonacute individuals, higher walkability may facilitate the use of nearby green spaces and thereby enhance potential health benefits. In contrast, among patients with acute CHD—who often experience greater functional and health limitations—the benefits of greenness may arise through alternative pathways, such as stress reduction or mitigation of harmful environmental exposures, which are less dependent on walkability. This interpretation is supported by previous evidence showing that neighborhood walkability was not predictive of outcomes among older adults following acute MI, suggesting that mobility limitations may attenuate the relevance of walkability in this population.^63^ Furthermore, prior evidence suggests that participation in structured rehabilitation programs may reduce reliance on the surrounding greenness for physical activity.^60^ Given that patients who experience an acute coronary event are more likely to participate in cardiac rehabilitation than patients with stable CHD,^64^ differential engagement in structured rehabilitation may provide an additional explanation for the modification of the greenness-mortality association by neighborhood walkability observed among stable, but not acute, CHD patients.

### Strengths and limitations

Several limitations of our study should be acknowledged. First, although NDVI objectively measures greenness, it cannot distinguish between specific types of green spaces, limiting our ability to determine which natural environments are most beneficial for cardiovascular health. Stratified analysis by walkability partially enabled us to examine the impact of urban parks separately, as dense urban settings in high-walkability areas typically offer urban parks rather than woodland or agricultural fields. Second, CHD status may have changed over time, as individuals classified as CHD-free at baseline could have developed CHD during follow-up, and stable CHD patients may have experienced acute events. Although such transitions are likely to occur over a long follow-up period, our use of baseline classification is consistent with prior epidemiological studies on chronic disease. In addition, the observed differences in associations across CHD groups suggest that any potential misclassification may not have fully obscured meaningful patterns. Third, the timing of follow-up initiation differed between individuals with and without CHD (ie, baseline survey interview vs hospitalization); however, our use of age as the time scale in Cox models and the averaging of long-term exposure may mitigate this concern. Fourth, the potential for residual confounding from unmeasured individual or contextual factors remains a concern. It should be noted that the contributing cohorts differ in demographic composition, cardiovascular risk factors, and contextual factors, reflecting their distinct designs and sampling frameworks. These differences, however, do not represent bias per se but rather the expected characteristics of population-based vs clinically defined cohorts. Our adjustment for harmonized key demographic and clinical characteristics, alongside contextual environmental variables, may partially address this concern. Importantly, differences across NDVI tertiles were consistent across CHD groups, implying a lower likelihood of differential confounding. In this regard, although comorbidities were assessed via interview in the population-based cohorts and via electronic medical records in the patient-based cohorts, this difference is unlikely to represent a major source of bias in the current study, given the good agreement previously reported between self-reported chronic conditions and corresponding medical record diagnoses.^65^^,^^66^ Fifth, it should be noted that, due to limitations in data availability and spatial coverage, participants lacking data on environmental covariates tended to reside in less urbanized areas, characterized by lower traffic-related air pollution and higher surrounding greenness. Consequently, the fully adjusted models may primarily reflect associations applicable to populations living in urban settings, where greenness is more likely to take the form of urban vegetation such as parks and street greenery. Importantly, this pattern did not differ substantially across CHD strata, supporting the comparability of results across CHD groups. Finally, generalizability should be considered when interpreting our findings, primarily regarding the environmental context in Israel, which is characterized by a high proportion of the population living in urban areas and relatively low levels of surrounding greenness compared with other countries.^61^^,^^67^^,^^68^ However, this limitation may also be viewed as a strength, since examining diverse populations across a wide range of environmental contexts is essential for understanding how exposure-response relationships vary across the full exposure spectrum. In this study, findings provide insight into the potential benefits of greenness in settings with limited availability of natural environments, where modest differences in exposure may be particularly relevant.

Our study has several key strengths, including its large sample size and broad population representation, covering both individuals with and without CHD. By assessing the association of greenness across different CHD profiles while ensuring a comparable exposure assessment, we provide a nuanced understanding of the potential cardiovascular benefits of green environments. In addition, incorporating comprehensive data on neighborhood walkability allowed us to better account for contextual-level confounding and investigate its role as a potential effect modifier. The study further benefits from an extensive follow-up period of up to 3 decades, along with a long record of high spatial resolution of greenness exposure.

## Conclusions

In this multicohort study of individuals with and without CHD, long-term exposure to greenness was more strongly and consistently associated with lower mortality among those with preexisting CHD than among individuals without CHD. The association was more pronounced among participants living in highly walkable neighborhoods. Our findings emphasize the relevance of accessible, walkable environments in strengthening the observed association between green space exposure and mortality among vulnerable populations and suggest that supportive environmental features may contribute to more favorable outcomes in secondary prevention contexts.

## Funding support and author disclosures

This work was supported by the 10.13039/501100003977Israel Science Foundation (10.13039/501100003977ISF, grant no. 2666/21; PI, Dr Gerber). The authors have reported that they have no relationships relevant to the contents of this paper to disclose.
