# Supplementary material for: Differential Association Between Surrounding Greenness and Mortality in Individuals With Coronary Heart Disease
Source: JACC Adv. 2026 Apr 9;5(5):102729. doi: 10.1016/j.jacadv.2026.102729 (PMC13091352; doi:10.1016/j.jacadv.2026.102729)
Supplement: Supplemental Materials [file mmc1.docx]

Supplementary materials for:

**Differential association between surrounding greenness and mortality in individuals with coronary heart disease**

*Calculation of walkability measures*

To capture differences in characteristics of the built environment, we calculated neighborhood walkability, defining the extent to which an area supports walking as a convenient, safe, and appealing mode of transportation and recreation.^1^ It is often defined based on land use patterns, transportation systems, and urban design, which reflects whether the neighborhood provides a pedestrian-friendly infrastructure, proximity to essential services and ease of accessing destinations without relying on a car.^2^ Although measures of neighborhood walkability have not been fully standardized, the measures of population density, land-use mix, and street connectivity have commonly been linked to health outcomes,^1^ including BMI,^3^ obesity-related mortality^4^ and cardiovascular mortality.^5^ Population density is commonly used as a walkability measure as it indicates the concentration of resources and infrastructure within a neighborhood, promoting accessibility and reducing reliance on cars.^6^ Street network connectivity is defined as the availability and the directness of alternative routes to get from one point to another in a street network. It is widely used as an indicator of walkability since better-connected streets provide shorter routes and better traffic flows.^2^ Land-use mix reflects the diversity of destinations within a given area facilitating access to services, which encourages walking; when measured within residential areas, a high land-use mix indicates that homes are in close proximity to occupational, retail and recreational areas, encouraging active transportation within the neighborhood.^2^ We obtained information on these three neighborhood components from several sources, and calculated them within 800 m buffer around participant’s home address via Geographic Information System (GIS) (ArcGIS Pro 2.8.0). *(i) Population density:* Data on population density was derived from the Israel Central Bureau of Statistics (ICBS) 2004 GIS layer (named: density_built_residence), which entails the number of residents divided by residential land-use area ratio for each statistical unit,^6^ and then computed mean population density within the buffer zone. *(ii)* *Street Connectivity:* We calculated street connectivity as the sum of street intersections within the buffer zone, while an intersection was identified as a point that connects three streets and above.^6^ To identify intersections, we used the roads GIS layer from [Open Street Map](https://www.openstreetmap.org/#map=8/31.438/35.074) 2019 and created nodes in the connecting points of at least three lines. *(iii)* *Land-use mix:* To capture neighborhood land-use diversity we used the Entropy Index, which is calculated based on the number and relative percentage of different land uses in the buffer area, according to the following formula:^7^


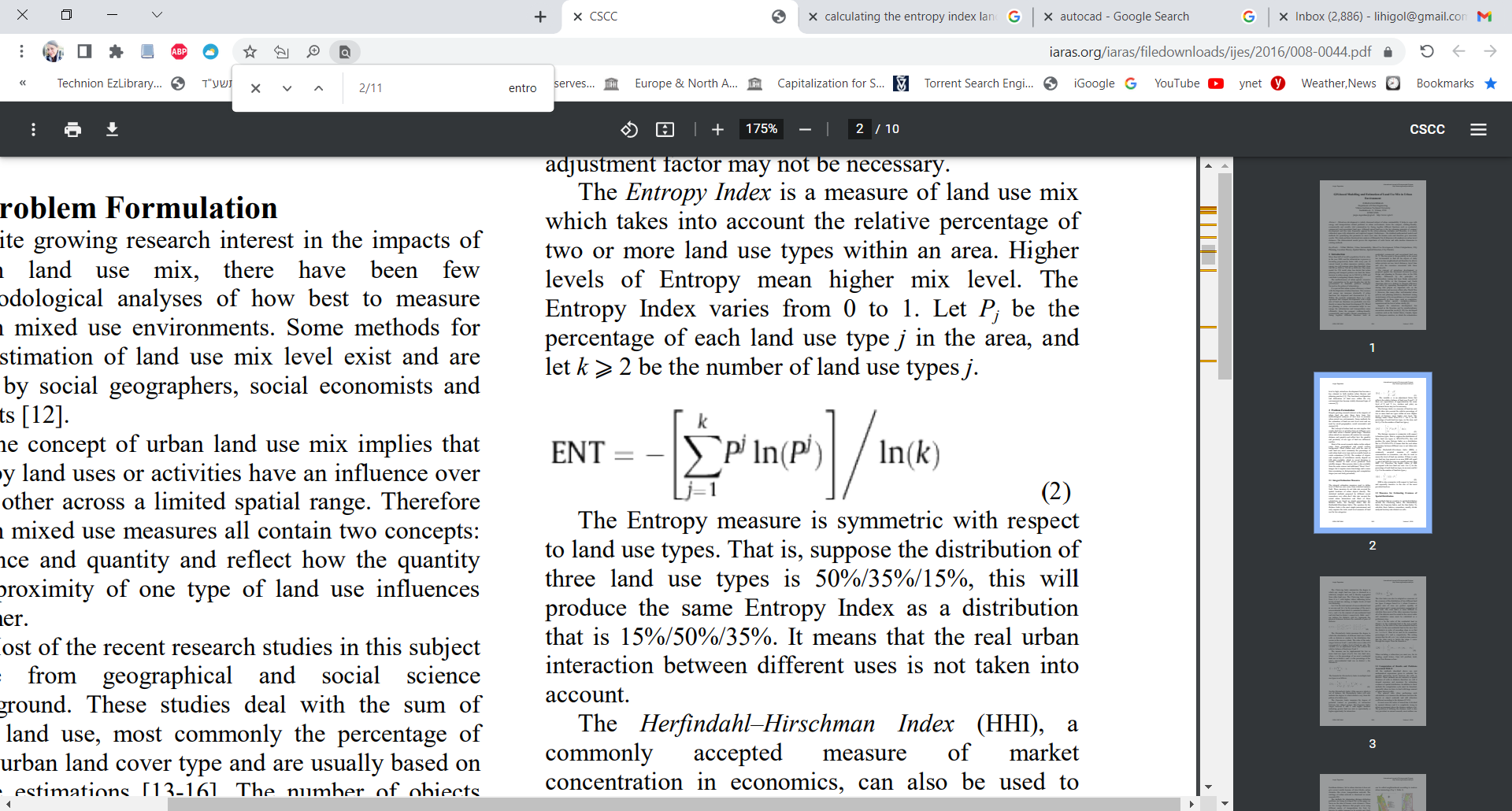


When *P^j^* denotes the percentage of each land use type *j* in the area and *k* ⩾ 2 is the number of land use types *j*. The entropy index varies from 0 to 1, with 0 indicating a homogeneous area containing one land use only, whereas a score of 1 indicates a heterogeneous area with all land use types equally distributed across the area. We extracted land-use data from the ICBS 2004 GIS layer, which includes 14 different land uses: education; health and welfare; public services; cultural, leisure, recreation and sports; commerce; industry and infrastructure; agricultural structures; residential; public open area; forest; plantations, orchards and olive groves; cultivated fields; other open area (Supplementary Figure S2). We calculated the entropy index based on the area for each land use within the buffer based on the given formula. *Calculation of the combined walkability construct:* To create a consolidated walkability index, we first calculated z-scores for the three components (population density, street connectivity and land-use mix) for each buffer size. Since connectivity strongly influences walkability by enhancing accessibility and ease of movement, we increased its weight by multiplying the z-score by 2 to reflect its greater impact on walkability. Finally, we summed the weighted z-scores of all components to derive the overall walkability index.^8^


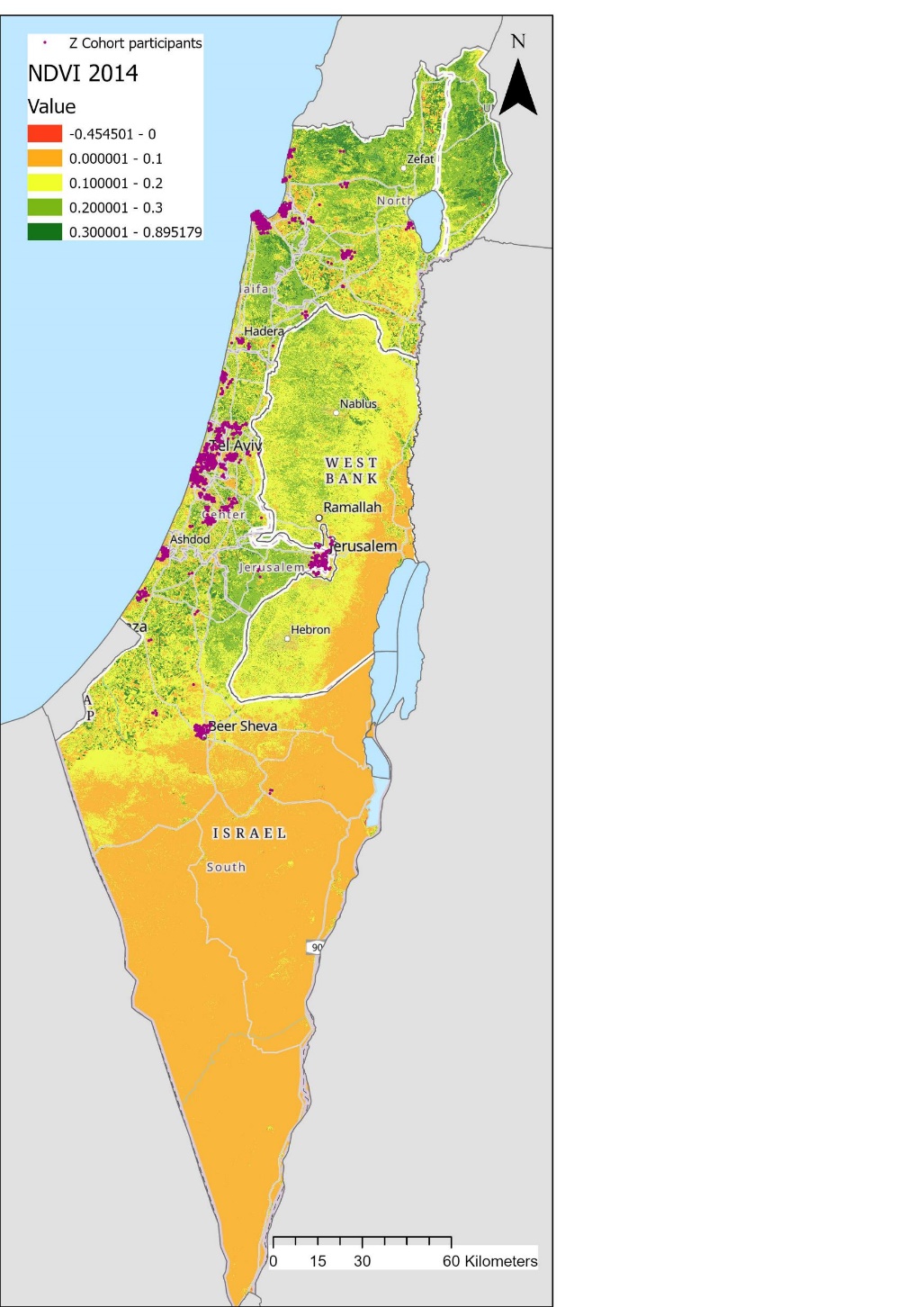


**Figure S1.** NDVI map of study area (based on 2014 data). Dots represent study participants.


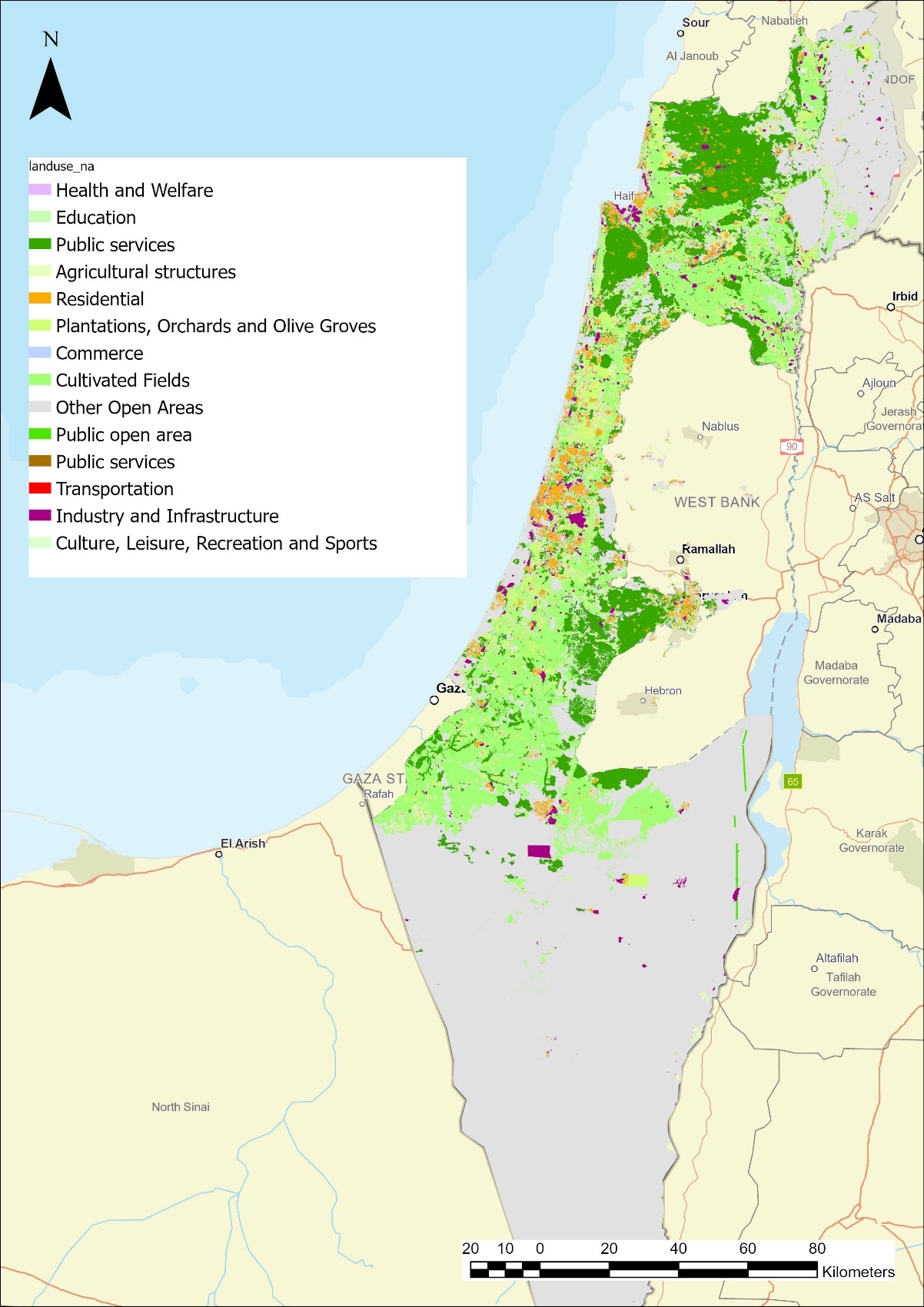


**Figure S2.** A map of land use data in Israel. Each color represents a different land use type of the 14 types included.

**
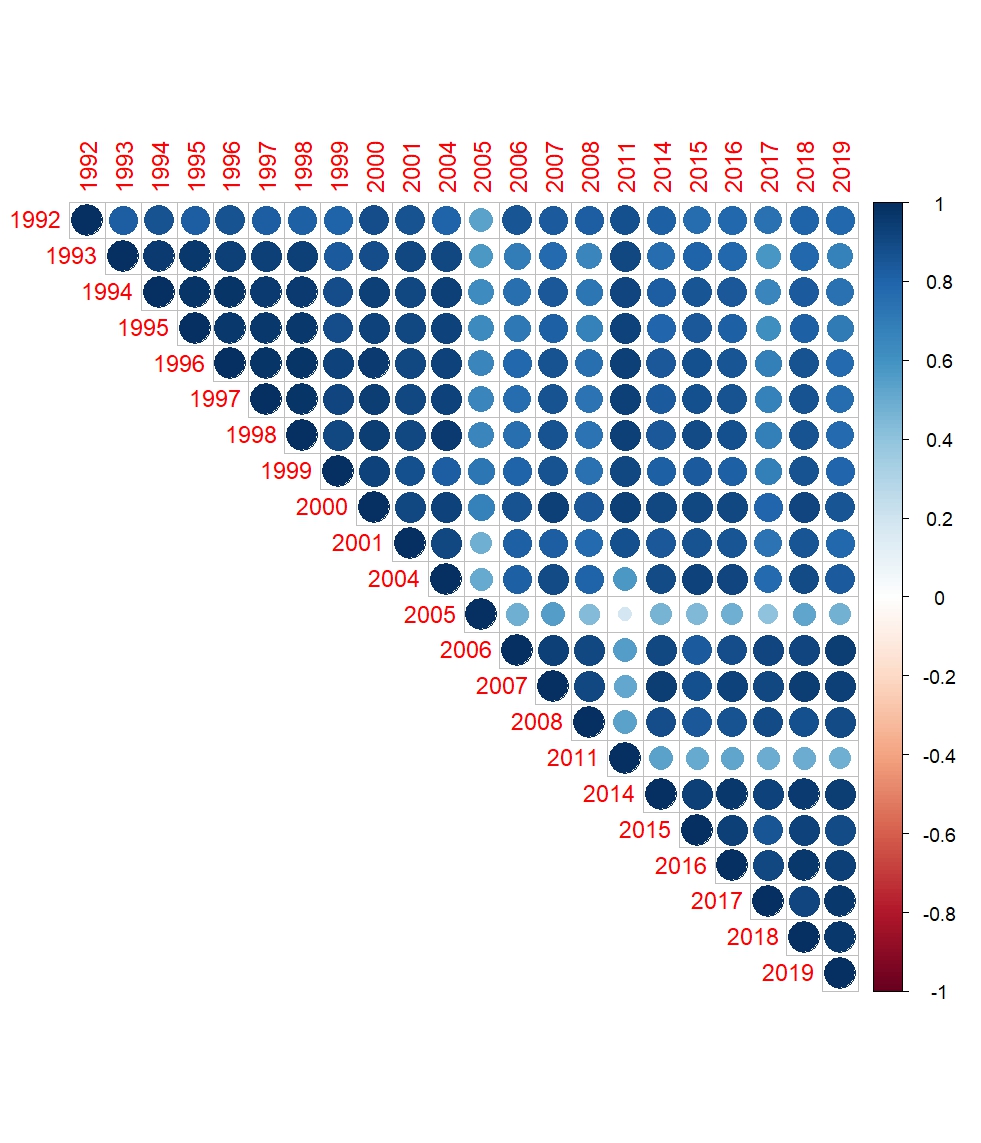
**

**Figure S3.** Correlation matrix between annual NDVI estimates in the pooled cohort.

**
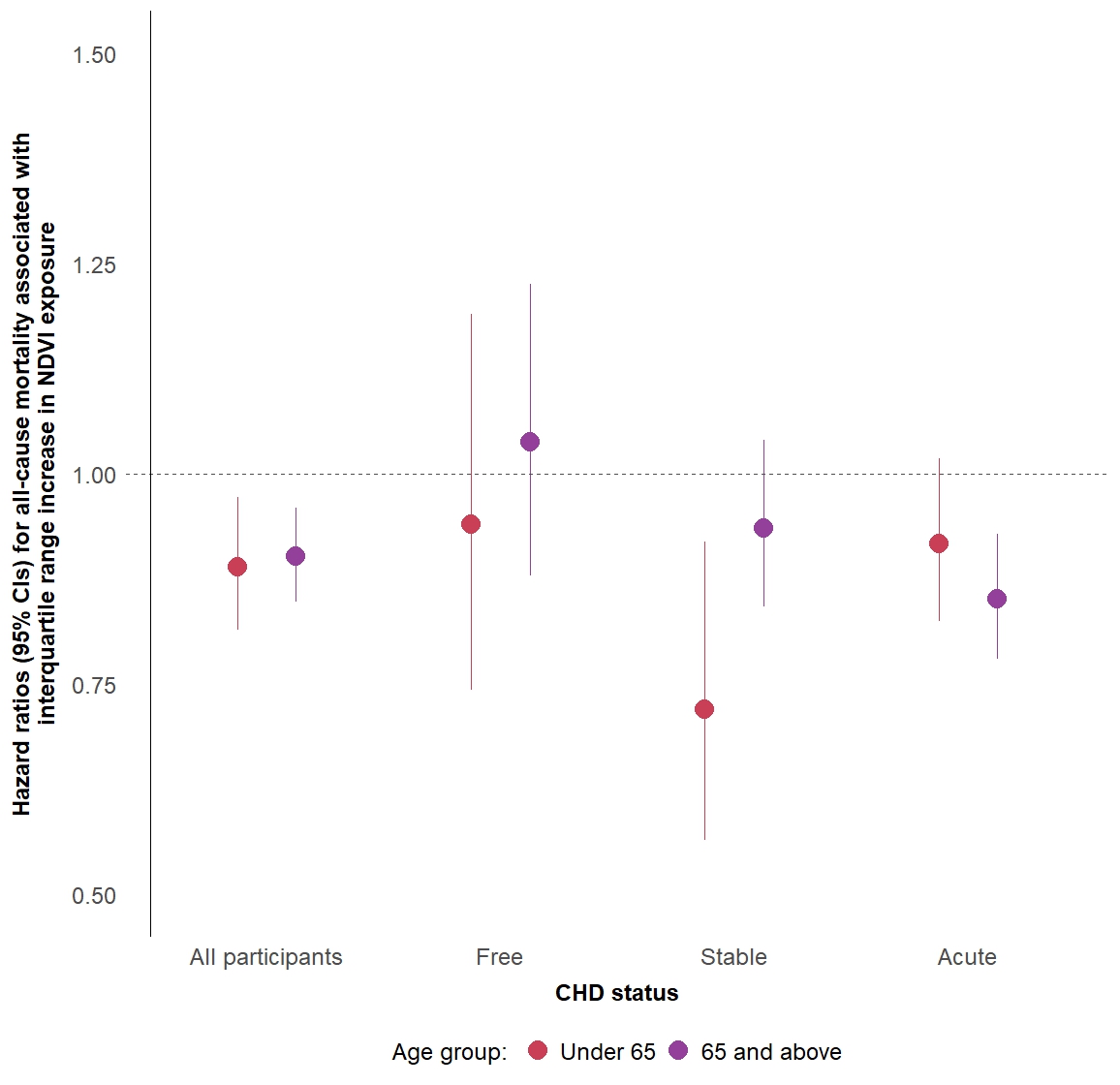
**

**Figure S4.** Association between greenness exposure, as indicated by NDVI in 800 m around residential location, and all-cause mortality, stratified by age group, among all participants, and by CHD status. HRs are presented for 1-IQR increase in NDVI (0.047). Models used age as the time scale, and were adjusted for sex, smoking, preexisting comorbidities (diabetes, hypertension and stroke), neighbourhood SES, year of study entry, living in an urban locality, exposure to traffic-related air pollution and walkability index. P-values for interaction were derived using likelihood ratio tests, comparing the full model with the NDVI*age group interaction term to a nested model without it. P-values for interaction: All participants, P>0.20; CHD-free, P>0.20; Stable CHD, P=0.09; Acute CHD, P=0.15. CI, confidence interval; HR, hazard ratio; IQR, interquartile range; NDVI, normalized difference vegetation index.

**
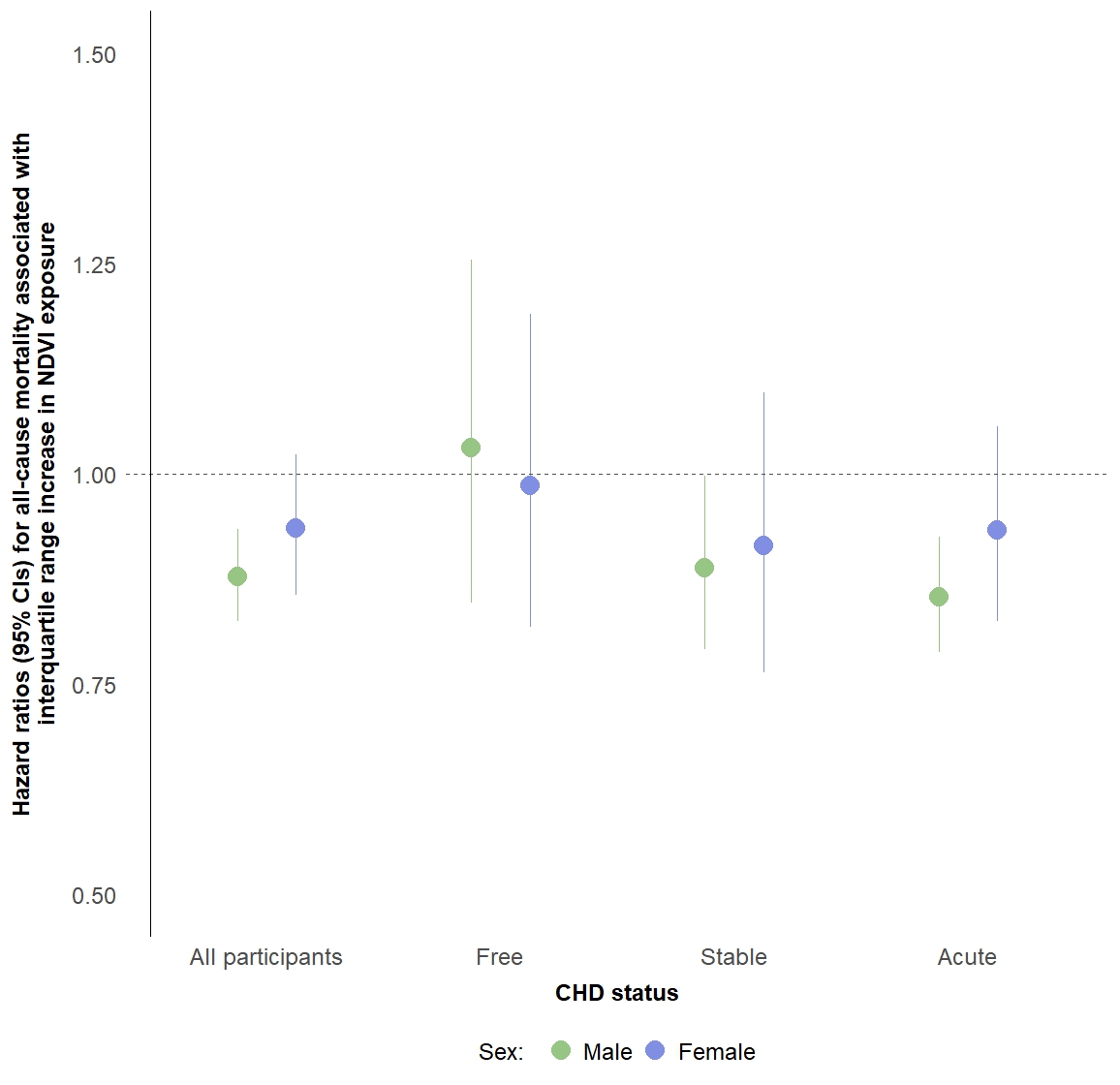
**

**Figure S5.** Association between greenness exposure, as indicated by NDVI in 800 m around residential location, and all-cause mortality, stratified by sex, among all participants, and by CHD status. HRs are presented for 1-IQR increase in NDVI (0.047). Models used age as the time scale, and were adjusted for smoking, preexisting comorbidities (diabetes, hypertension and stroke), neighbourhood SES, year of study entry, living in an urban locality, exposure to traffic-related air pollution and walkability index. P-values for interaction were derived using likelihood ratio tests, comparing the full model with the NDVI*sex interaction term to a nested model without it. P-values for interaction: All participants, P=0.15; CHD-free, P>0.20; Stable CHD, P>0.20; Acute CHD, P=0.15. CI, confidence interval; HR, hazard ratio; IQR, interquartile range; NDVI, normalized difference vegetation index.

**
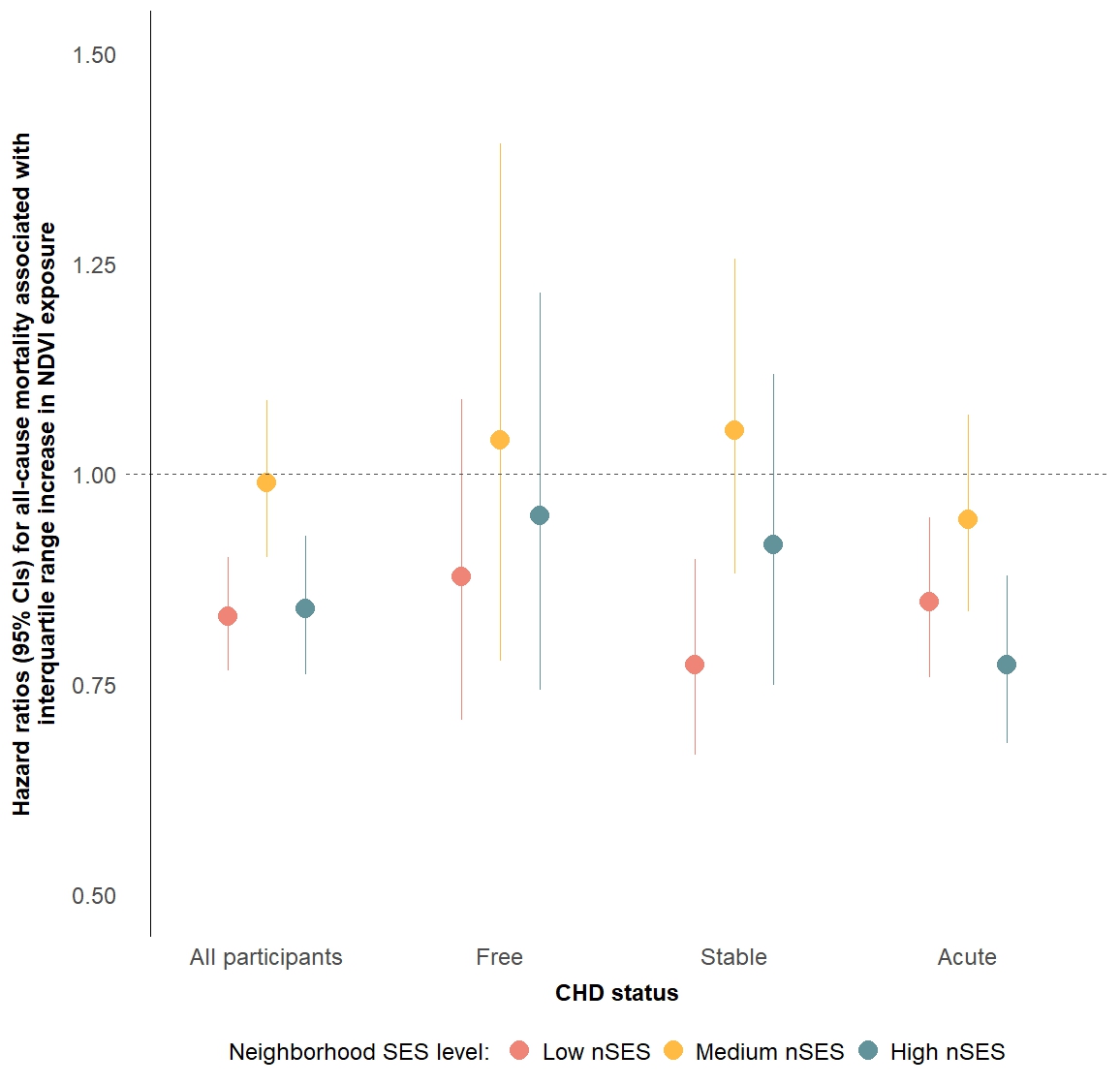
**

**Figure S6.** Association between greenness exposure, as indicated by NDVI in 800 m around residential location, and all-cause mortality, stratified by neighborhood SES tertile, among all participants, and by CHD status. HRs are presented for 1-IQR increase in NDVI (0.047). Models used age as the time scale, and were adjusted for sex, smoking, preexisting comorbidities (diabetes, hypertension and stroke), year of study entry, living in an urban locality, exposure to traffic-related air pollution and walkability index. P-values for interaction were derived using likelihood ratio tests, comparing the full model with the NDVI*SES interaction term to a nested model without it. P-values for interaction: All participants, P=0.008; CHD-free, P=0.14; Stable CHD, P>0.20; Acute CHD, P=0.02. CI, confidence interval; HR, hazard ratio; IQR, interquartile range; NDVI, normalized difference vegetation index; SES, socioeconomic status.

**Table S1. Detailed description of satellite-based data obtained in the current study.**

| **Date Acquired South** | **Date Acquired Center** | **Date Acquired North** | **Satellite** | **Year** |
| --- | --- | --- | --- | --- |
| 23.05.1992 | 23.05.1992 | 23.05.1992 | Landsat 5 | 1992 |
| 23.03.1993 | 23.03.1993 | 23.03.1993 | Landsat 5 | 1993 |
| 13.05.1994 | 11.04.1995 | 11.04.1994 | Landsat 5 | 1994 |
| 29.03.1995 | 29.03.1995 | 29.03.1995 | Landsat 5 | 1995 |
| 16.04.1996 | 16.04.1996 | 16.04.1996 | Landsat 5 | 1996 |
| 19.04.1997 | 19.04.1997 | 19.04.1997 | Landsat 5 | 1997 |
| 06.04.1998 | 06.04.1998 | 06.04.1998 | Landsat 5 | 1998 |
| 25.04.1999 | 25.04.1999 | 25.04.1999 | Landsat 5 | 1999 |
| 27.04.2000 | 27.04.2000 | 27.04.2000 | Landsat 5 | 2000 |
| 29.03.2001 | 29.03.2001 | Missing | Landsat 5 | 2001 |
| Invalid | Invalid | Invalid | Landsat 5 | 2002 |
| Missing | Missing | Missing | Landsat 5 | 2003 |
| 21.03.2004 | 21.03.2004 | 21.03.2004 | Landsat 5 | 2004 |
| 25.04.2005 | 25.04.2005 | 25.04.2005 | Landsat 5 | 2005 |
| 14.05.2006 | 14.05.2006 | 14.05.2006 | Landsat 5 | 2006 |
| 01.05.2007 | 01.05.2007 | 01.05.2007 | Landsat 5 | 2007 |
| 03.05.2008 | 03.05.2008 | 03.05.2008 | Landsat 5 | 2008 |
| 20.04.2009 | 20.04.2009 | Missing | Landsat 5 | 2009 |
| 07.04.2010 | Missing | Missing | Landsat 5 | 2010 |
| 10.04.2011 | 17.04.2011 west | 17.04.2011 west | Landsat 5 | 2011 |
| Missing | Missing | Missing | No Landsat data available | 2012 |
| 01.05.2013 | 01.05.2013 east | 21.03.2013 | Landsat 8 | 2013 |
| 18.04.2014 | 18.04.2014 | 18.04.2014 | Landsat 8 | 2014 |
| 05.04.2015 | 05.04.2015 | 05.04.2015 | Landsat 8 | 2015 |
| 07.04.2016 | 07.04.2016 | 07.04.2016 | Landsat 8 | 2016 |
| 12.05.2017 | 12.05.2017 | 12.05.2017 | Landsat 8 | 2017 |
| 13.04.2018 | 13.04.2018 | 13.04.2018 | Landsat 8 | 2018 |
| 18.05.2019 | 18.05.2019 | 18.05.2019 | Landsat 8 | 2019 |

**Table S2. Comparison of characteristics between included and excluded participants**

| Inclusion status | Excluded | Included | P | SMD | Missing | |
| --- | --- | --- | --- | --- | --- | --- |
| N | 1,922 | 17,431 |  |  | **Excluded** | **Included** |
| Age, years, mean ± SD | 64.0 (15.0) | 63.6 (15.3) | 0.25 | 0.03 | 0 (0.0) | 0 (0.0) |
| Female sex, n (%) | 704 (36.6) | 5,286 (30.3) | <0.01 | 0.13 | 0 (0.0) | 0 (0.0) |
| Neighbourhood SES, mean ± SD | 11.0 (4.5) | 11.5 (4.0) | <0.01 | 0.12 | 1,083 (56.4) | 1,299 (7.5) |
| Arab ethnicity, n (%) | 217 (11.3) | 1821 (10.4) | 0.26 | 0.03 | 5 (0.3) | 5 (0.03) |
| Smoking, n (%) | 515 (27.1) | 5,908 (33.9) | <0.01 | 0.15 | 20 (1.0) | 10 (0.1) |
| Diabetes, n (%) | 611 (32.2) | 6,002 (34.4) | 0.05 | 0.05 | 23 (1.2) | 8 (0.1) |
| Hypertension, n (%) | 1,075 (57.2) | 10,344 (59.7) | 0.04 | 0.05 | 41 (2.1) | 102 (0.6) |
| Stroke, n (%) | 112 (5.9) | 818 (4.7) | 0.02 | 0.05 | 31 (1.6) | 9 (0.1) |
| Nitrogen oxides, ppb, mean ± SD | 22.0 (11.4) | 25.4 (9.7) | <0.01 | 0.32 | 1,140 (59.3) | 976 (5.6) |
| Walkability index, mean ± SD | 0.0 (2.7) | 0.1 (2.6) | 0.81 | 0.01 | 1,140 (59.3) | 1,463 (8.4) |
| NDVI, mean ± SD | 0.122 (0.030) | 0.125 (0.035) | 0.03 | 0.08 | 1,136 (59.1) | 0 (0.0) |

SMDs are reported to enable interpretable comparison of group differences across variables. Reported SMDs represent the average of all pairwise comparisons between included and excluded participants. NDVI, normalized difference vegetation index; ppb, parts per billion; SD, standard deviation; SES, socioeconomic status, SMD, standardized mean difference.

**Table S3. Cohorts’ characteristics by CHD status**

| CHD status |  | Free of CHD | Stable CHD | Acute CHD | P | SMD |
| --- | --- | --- | --- | --- | --- | --- |
| N |  | 3,959 | 4,771 | 8,701 |  |  |
| Age, years, mean ± SD |  | 51.4 (17.5) | 69.6 (11.3) | 65.7 (13.0) | <0.01 | 0.83 |
| Female sex, n (%) |  | 2,151 (54.3) | 1,109 (23.2) | 2,026 (23.3) | <0.01 | 0.45 |
| Neighbourhood SES, mean ± SD |  | 10.6 (4.7) | 11.4 (3.9) | 11.9 (3.7) | <0.01 | 0.21 |
| Arab ethnicity, n (%) |  | 955 (24.2) | 388 (8.1) | 478 (5.5) | <0.01 | 0.37 |
| Smoking, n (%) |  | 979 (24.8) | 1,340 (28.1) | 3,589 (41.2) | <0.01 | 0.24 |
| Diabetes, n (%) |  | 430 (10.9) | 2,267 (47.5) | 3,305 (38.0) | <0.01 | 0.58 |
| Hypertension, n (%) |  | 920 (23.9) | 3,735 (78.3) | 5,689 (65.4) | <0.01 | 0.84 |
| Stroke, n (%) |  | 90 (2.3) | 230 (4.8) | 498 (5.7) | <0.01 | 0.12 |
| Nitrogen oxides, ppb, mean ± SD |  | 22.3 (11.1) | 24.8 (9.3) | 27.2 (8.6) | <0.01 | 0.33 |
| Walkability level, n (%) |  |  |  |  | <0.01 | 0.20 |
| Low |  | 1,311 (33.9) | 1,588 (37.5) | 2,419 (30.8) |  |  |
| Medium |  | 1,046 (27.0) | 1,489 (35.2) | 2,797 (35.6) |  |  |
| High |  | 1,514 (39.1) | 1,154 (27.3) | 2,650 (33.7) |  |  |
| Living in urban locality, n (%) |  | 3,389 (88.6) | 3,933 (83.9) | 7,785 (90.6) | <0.01 | 0.13 |
| Mean (range) NDVI |  | 0.127  (0.016, 0.273) | 0.128  (0.016, 0.279) | 0.123  (0.024, 0.277) | <0.01 | 0.10 |

SMDs are reported to enable interpretable comparison of group differences across variables. Reported SMDs represent the average of all pairwise comparisons across CHD status groups. CHD, coronary heart disease; NDVI, normalized difference vegetation index; ppb, parts per billion; SD, standard deviation; SES, socioeconomic status, SMD, standardized mean difference.

| CHD status | MABAT First | MABAT Zahav | MI Cohort | PCI Cohort | P | SMD |
| --- | --- | --- | --- | --- | --- | --- |
| N | 2,829 | 1,130 | 1,428 | 12,044 |  |  |
| Age, years, mean ± SD | 42.3 (11.0) | 74.3 (6.2) | 53.9 (8.2) | 68.7 (12.1) | <0.01 | 1.98 |
| Female sex, n (%) | 1,500 (53.0) | 651 (57.6) | 272 (19.0) | 2,863 (23.8) | <0.01 | 0.53 |
| Neighbourhood SES, mean ± SD | 10.1 (4.6) | 11.8 (4.7) | 12.5 (3.8) | 11.6 (3.7) | <0.01 | 0.30 |
| Arab ethnicity, n (%) | 766 (27.1) | 189 (16.8) | 23 (1.6) | 843 (7.0) | <0.01 | 0.45 |
| Smoking, n (%) | 851 (30.1) | 128 (11.4) | 742 (52.0) | 4,187 (34.8) | <0.01 | 0.49 |
| Diabetes, n (%) | 159 (5.6) | 271 (24.0) | 349 (24.4) | 5,223 (43.4) | <0.01 | 0.48 |
| Hypertension, n (%) | 339 (12.4) | 581 (52.1) | 550 (38.5) | 8,874 (73.7) | <0.01 | 0.77 |
| Stroke, n (%) | 14 (0.5) | 76 (6.7) | 36 (2.5) | 692 (5.7) | <0.01 | 0.20 |
| Nitrogen oxides, ppb, mean ± SD | 21.7 (11.0) | 23.9 (11.4) | 30.4 (9.4) | 25.8 (8.7) | <0.01 | 0.46 |
| Walkability level, n (%) |  |  |  |  | <0.01 | 0.13 |
| Low | 930 (33.9) | 381 (33.7) | 453 (32.4) | 3,554 (33.2) |  |  |
| Medium | 726 (26.5) | 320 (28.3) | 450 (32.2) | 3,836 (35.8) |  |  |
| High | 1,086 (39.6) | 428 (37.9) | 493 (35.3) | 3,311 (30.9) |  |  |
| Living in urban locality, n (%) | 2,259 (83.8) | 1,130 (100.0) | 1,331 (94.4) | 10,387 (87.5) | <0.01 | 0.37 |
| Mean (range) NDVI | 0.128  (0.016-0.273) | 0.123  (0.030-0.223) | 0.117  (0.024-0.237) | 0.126  (0.160-0.279) | <0.01 | 0.19 |

**Table S4. Characteristics of the four contributing cohorts**

MABAT First and MABAT Zahav are population-based national health and nutrition survey cohorts of adults aged 25–64 years (1999–2001) and ≥65 years (2005–2006), respectively; MI cohort is a patient-based cohort of individuals aged ≤65 years hospitalized for first incident myocardial infarction in central Israel (1992–1993); and PCI is an administrative cohort of consecutive patients undergoing percutaneous coronary intervention at Rabin Medical Center (2004–2014). SMDs are reported to enable interpretable comparison of group differences across variables. Reported SMDs represent the average of all pairwise comparisons across CHD status groups. CHD, coronary heart disease; NDVI, normalized difference vegetation index; ppb, parts per billion; SD, standard deviation; SES, socioeconomic status, SMD, standardized mean difference.

**Table S5. Comparison of characteristics between participants with and without neighborhood SES data across CHD categories**

|  | **Free of CHD**  **(n=3,959)** | | | **Stable CHD**  **(n=4,771)** | | | **Acute CHD**  **(n=8,701)** | | |
| --- | --- | --- | --- | --- | --- | --- | --- | --- | --- |
|  | **Non-missing** | **Missing** | **SMD** | **Non-missing** | **Missing** | **SMD** | **Non-missing** | **Missing** | **SMD** |
| n | 3798 | 161 |  | 4293 | 478 |  | 8041 | 660 |  |
| Age, years, mean ± SD | 51.8 (17.5) | 42.0 (12.5) | 0.65 | 69.7 (11.3) | 68.8 (11.6) | 0.08 | 65.9 (13.1) | 64.1 (11.8) | 0.14 |
| Female sex, n (%) | 2075 (54.6) | 76 (47.2) | 0.15 | 1035 (24.1) | 74 (15.5) | 0.22 | 1905 (23.7) | 121 (18.3) | 0.13 |
| Arab ethnicity, n (%) | 937 (24.7) | 18 (11.2) | 0.36 | 383 (8.9) | 5 (1.0) | 0.37 | 470 (5.8) | 8 (1.2) | 0.25 |
| Smoking, n (%) | 936 (24.7) | 43 (26.7) | 0.05 | 1232 (28.7) | 108 (22.6) | 0.14 | 3345 (41.6) | 244 (37.0) | 0.09 |
| Diabetes, n (%) | 420 (11.1) | 10 (6.2) | 0.17 | 2058 (47.9) | 209 (43.7) | 0.08 | 3075 (38.2) | 230 (34.8) | 0.07 |
| Hypertension, n (%) | 900 (24.3) | 20 (12.7) | 0.3 | 3377 (78.7) | 358 (74.9) | 0.09 | 5281 (65.7) | 408 (61.8) | 0.08 |
| Stroke, n (%) | 90 (2.4) | 0 (0.0) | 0.22 | 212 (4.9) | 18 (3.8) | 0.06 | 470 (5.8) | 28 (4.2) | 0.07 |
| Nitrogen oxides, ppb, mean ± SD | 22.5 (11.2) | 18.9 (8.6) | 0.36 | 25.2 (9.3) | 20.2 (8.1) | 0.57 | 27.4 (8.5) | 23.2 (8.9) | 0.49 |
| Walkability index, mean ± SD | 0.0 (2.8) | 1.5 (4.0) | 0.42 | 0.0 (2.5) | -1.5 (2.9) | 0.55 | 0.3 (2.4) | -1.6 (2.8) | 0.71 |
| Living in urban locality, n (%) | 3335 (90.7) | 54 (36.0) | 1.38 | 3882 (90.9) | 51 (12.2) | 2.55 | 7694 (96.1) | 91 (15.6) | 2.76 |
| NDVI, mean ± SD | 0.1 (0.0) | 0.2 (0.0) | 1.17 | 0.1 (0.0) | 0.2 (0.0) | 1.48 | 0.1 (0.0) | 0.2 (0.0) | 1.49 |

Reported SMDs represent the average of all pairwise comparisons in each CHD status group. CHD, coronary heart disease; NDVI, normalized difference vegetation index; ppb, parts per billion; SD, standard deviation; SES, socioeconomic status; SMD, standardized mean difference.

**Table S6. Comparison of characteristics between participants with and without NO_x_ levels data across CHD categories**

|  | **Free of CHD**  **(n=3,959)** | | | **Stable CHD**  **(n=4,771)** | | | **Acute CHD**  **(n=8,701)** | | |
| --- | --- | --- | --- | --- | --- | --- | --- | --- | --- |
|  | **Non-missing** | **Missing** | **SMD** | **Non-missing** | **Missing** | **SMD** | **Non-missing** | **Missing** | **SMD** |
| n | 3928 | 31 |  | 4486 | 285 |  | 8041 | 660 |  |
| Age, years, mean ± SD | 51.5 (17.5) | 46.3 (16.5) | 0.31 | 69.9 (11.3) | 65.9 (11.3) | 0.35 | 65.9 (13.2) | 64.2 (11.5) | 0.13 |
| Female sex, n (%) | 2135 (54.4) | 16 (51.6) | 0.05 | 1046 (23.3) | 63 (22.1) | 0.03 | 1892 (23.5) | 134 (20.3) | 0.08 |
| Neighbourhood SES, mean ± SD | 10.6 (4.7) | 9.5 (4.6) | 0.24 | 11.4 (4.0) | 11.8 (1.8) | 0.13 | 11.9 (3.7) | 11.2 (1.9) | 0.25 |
| Arab ethnicity, n (%) | 950 (24.2) | 5 (16.7) | 0.19 | 388 (8.6) | 0 (0.0) | 0.44 | 476 (5.9) | 2 (0.3) | 0.33 |
| Smoking, n (%) | 969 (24.7) | 10 (32.3) | 0.17 | 1233 (27.5) | 107 (37.5) | 0.22 | 3263 (40.6) | 326 (49.4) | 0.18 |
| Diabetes, n (%) | 429 (10.9) | 1 (3.2) | 0.3 | 2153 (48.0) | 114 (40.0) | 0.16 | 3066 (38.1) | 239 (36.2) | 0.04 |
| Hypertension, n (%) | 914 (23.9) | 6 (19.4) | 0.11 | 3507 (78.2) | 228 (80.0) | 0.04 | 5244 (65.2) | 445 (67.4) | 0.05 |
| Stroke, n (%) | 90 (2.3) | 0 (0.0) | 0.22 | 216 (4.8) | 14 (4.9) | <0.01 | 470 (5.8) | 28 (4.2) | 0.07 |
| Walkability index, mean ± SD | 0.0 (2.9) | -0.9 (1.8) | 0.37 | -0.1 (2.5) | -0.7 (2.2) | 0.25 | 0.2 (2.5) | -0.6 (2.2) | 0.36 |
| Living in urban locality, n (%) | 3365 (88.6) | 24 (77.4) | 0.3 | 3724 (84.4) | 209 (76.8) | 0.19 | 7362 (92.3) | 423 (68.3) | 0.63 |
| NDVI, mean ± SD | 0.1 (0.0) | 0.1 (0.0) | 1.1 | 0.1 (0.0) | 0.1 (0.1) | 0.57 | 0.1 (0.0) | 0.1 (0.1) | 0.11 |

Reported SMDs represent the average of all pairwise comparisons in each CHD status group. CHD, coronary heart disease; NDVI, normalized difference vegetation index; SD, standard deviation; SES, socioeconomic status; SMD, standardized mean difference.

**Table S7. Comparison of characteristics between participants with and without NO_x_ levels data across CHD categories**

|  | **Free of CHD**  **(n=3,959)** | | | **Stable CHD**  **(n=4,771)** | | | **Acute CHD**  **(n=8,701)** | | |
| --- | --- | --- | --- | --- | --- | --- | --- | --- | --- |
|  | **Non-missing** | **Missing** | **SMD** | **Non-missing** | **Missing** | **SMD** | **Non-missing** | **Missing** | **SMD** |
| n | 3871 | 88 |  | 4231 | 540 |  | 7866 | 835 |  |
| Age, years, mean ± SD | 51.6 (17.5) | 42.6 (11.0) | 0.62 | 70.1 (11.2) | 65.9 (11.9) | 0.36 | 66.0 (13.1) | 63.1 (12.2) | 0.23 |
| Female sex, n (%) | 2108 (54.5) | 43 (48.9) | 0.11 | 1012 (23.9) | 97 (18.0) | 0.15 | 1879 (23.9) | 147 (17.6) | 0.16 |
| Neighbourhood SES, mean ± SD | 10.6 (4.7) | 11.7 (3.8) | 0.25 | 11.5 (3.9) | 10.6 (3.9) | 0.23 | 11.9 (3.7) | 11.6 (3.8) | 0.1 |
| Arab ethnicity, n (%) | 943 (24.4) | 12 (13.6) | 0.28 | 354 (8.4) | 34 (6.3) | 0.08 | 421 (5.4) | 57 (6.8) | 0.06 |
| Smoking, n (%) | 956 (24.8) | 23 (26.1) | 0.03 | 1161 (27.4) | 179 (33.1) | 0.12 | 3215 (40.9) | 374 (44.8) | 0.08 |
| Diabetes, n (%) | 425 (11.0) | 5 (5.7) | 0.19 | 2022 (47.8) | 245 (45.4) | 0.05 | 3020 (38.4) | 285 (34.1) | 0.09 |
| Hypertension, n (%) | 911 (24.2) | 9 (10.3) | 0.37 | 3344 (79.0) | 391 (72.4) | 0.16 | 5196 (66.1) | 493 (59.0) | 0.15 |
| Stroke, n (%) | 90 (2.3) | 0 (0.0) | 0.22 | 198 (4.7) | 32 (5.9) | 0.06 | 455 (5.8) | 43 (5.1) | 0.03 |
| Nitrogen oxides, ppb, mean ± SD | 22.5 (11.2) | 15.7 (6.7) | 0.73 | 25.1 (9.3) | 22.4 (9.5) | 0.28 | 27.4 (8.6) | 25.0 (8.9) | 0.27 |
| Living in urban locality, n (%) | 3382 (90.3) | 7 (8.8) | 2.81 | 3692 (88.3) | 241 (47.5) | 0.97 | 7293 (93.5) | 492 (62.4) | 0.81 |
| NDVI, mean ± SD | 0.1 (0.0) | 0.2 (0.0) | 1.89 | 0.1 (0.0) | 0.2 (0.0) | 0.7 | 0.1 (0.0) | 0.1 (0.0) | 0.59 |

Reported SMDs represent the average of all pairwise comparisons in each CHD status group. CHD, coronary heart disease; NDVI, normalized difference vegetation index; ppb, parts per billion ; SD, standard deviation; SES, socioeconomic status; SMD, standardized mean difference.

**Table S8. Cohort characteristics by CHD status and greenness exposure**

|  | Free of CHD  (n=3,959) | | | | Stable CHD  (n=4,771) | | | | Acute CHD  (n=8,701) | | | |
| --- | --- | --- | --- | --- | --- | --- | --- | --- | --- | --- | --- | --- |
| NDVI tertiles | **Low** | **Medium** | **High** | **SMD** | **Low** | **Medium** | **High** | **SMD** | **Low** | **Medium** | **High** | **SMD** |
| Mean (range) NDVI | 0.093  (0.016–0.107) | 0.121  (0.107–0.137) | 0.166  (0.137–0.273) |  | 0.087  (0.016–0.107) | 0.123  (0.107–0.137) | 0.167  (0.137–0.28) |  | 0.089  (0.024–0.107) | 0.122  (0.107–0.137) | 0.164  (0.137–0.277) |  |
| n | 1,217 | 1,474 | 1,268 |  | 1,440 | 1,595 | 1,736 |  | 3,154 | 2,742 | 2,805 |  |
| Age, years, mean ± SD | 51.5 (17.8) | 52.2 (17.2) | 50.5 (17.4) | 0.07 | 71.2 (11.1) | 68.8 (11.5) | 69.1 (11.3) | 0.14 | 66.4 (13.5) | 65.7 (12.9) | 65.0 (12.7) | 0.07 |
| Female sex, n (%) | 674 (55.4) | 812 (55.1) | 665 (52.4) | 0.04 | 381 (26.5) | 369 (23.1) | 359 (20.7) | 0.09 | 816 (25.9) | 615 (22.4) | 595 (21.2) | 0.07 |
| Neighbourhood SES, mean ± SD | 9.7 (4.2) | 10.9 (4.7) | 11.2 (5.0) | 0.22 | 11.3 (3.3) | 11.2 (4.3) | 11.7 (4.0) | 0.09 | 11.2 (3.4) | 12.5 (3.6) | 12.3 (3.9) | 0.24 |
| Arab ethnicity, n (%) | 352 (28.9) | 314 (21.3) | 289 (22.8) | 0.12 | 93 (6.5) | 209 (13.1) | 86 (5.0) | 0.19 | 231 (7.3) | 150 (5.5) | 97 (3.5) | 0.12 |
| Smoking, n (%) | 315 (25.9) | 341 (23.3) | 323 (25.5) | 0.04 | 396 (27.5) | 466 (29.2) | 478 (27.5) | 0.03 | 1,329 (42.1) | 1,118 (40.8) | 1,142 (40.7) | 0.02 |
| Diabetes, n (%) | 144 (11.8) | 163 (11.1) | 123 (9.7) | 0.05 | 677 (47.0) | 749 (47.0) | 841 (48.4) | 0.02 | 1,222 (38.7) | 1,004 (36.6) | 1,079 (38.5) | 0.03 |
| Hypertension, n (%) | 281 (23.5) | 351 (24.5) | 288 (23.5) | 0.02 | 1,153 (80.1) | 1,247 (78.2) | 1,335 (76.9) | 0.05 | 2,108 (66.8) | 1,776 (64.8) | 1,805 (64.3) | 0.03 |
| Stroke, n (%) | 31 (2.5) | 36 (2.5) | 23 (1.8) | 0.03 | 82 (5.7) | 70 (4.4) | 78 (4.5) | 0.04 | 205 (6.5) | 154 (5.6) | 139 (5.0) | 0.04 |
| Nitrogen oxides, ppb, mean ± SD | 27.3 (11.9) | 22.7 (10.4) | 17.1 (8.5) | 0.67 | 29.4 (9.0) | 25.2 (9.1) | 20.7 (7.9) | 0.68 | 30.1 (8.8) | 27.4 (8.0) | 23.4 (7.5) | 0.55 |
| Walkability level, n (%) |  |  |  | 0.45 |  |  |  | 0.47 |  |  |  | 0.44 |
| Low | 269 (22.2) | 449 (30.6) | 593 (49.9) |  | 299 (22.3) | 581 (39.3) | 708 (50.2) |  | 576 (19.5) | 760 (29.9) | 1,083 (45.8) |  |
| Medium | 318 (26.2) | 418 (28.5) | 310 (26.1) |  | 473 (35.2) | 539 (36.4) | 477 (33.8) |  | 1052 (35.5) | 932 (36.7) | 813 (34.3) |  |
| High | 627 (51.6) | 602 (41.0) | 285 (24.0) |  | 570 (42.5) | 359 (24.3) | 225 (16.0) |  | 1333 (45.0) | 846 (33.3) | 471 (19.9) |  |
| Living in urban locality, n (%) | 1,190 (97.9) | 1,363 (95.2) | 836 (70.9) | 0.54 | 1,413 (98.3) | 1,513 (95.0) | 1,007 (60.8) | 0.71 | 3132 (99.4) | 2,684 (98.1) | 1,969 (72.8) | 0.57 |

Reported SMDs represent the average of all pairwise comparisons across NDVI tertiles in each CHD status group. CHD, coronary heart disease; NDVI, normalized difference vegetation index; ppb, parts per billion; SD, standard deviation; SES, socioeconomic status; SMD, standardized mean difference.

**Table S9. Association between 800-m greenness exposure using alternative exposure matrices and all-cause mortality by CHD status**

|  | Free of CHD | | | Stable CHD | | | Acute CHD | | |
| --- | --- | --- | --- | --- | --- | --- | --- | --- | --- |
|  | **Model 1^a^** | **Model 2^b^** | **Model 3^c^** | **Model 1^a^** | **Model 2^b^** | **Model 3^c^** | **Model 1^a^** | **Model 2^b^** | **Model 3^c^** |
| Trimmed mean | | | | | | | | | |
|  | n=3,959, 820 events | | | n=4,771, 1,824 events | | | n=8,701, 3,411 events | | |
|  | 0.87  (0.78, 0.96) | 1.00  (0.90, 1.12) | 0.99  (0.87, 1.13) | 0.99  (0.93, 1.05) | 0.99  (0.93, 1.06) | 0.90  (0.82, 1.00) | 0.84  (0.80, 0.88) | 0.87  (0.82, 0.91) | 0.87  (0.82, 0.94) |
| *** P_for interaction_ < 0.001 | | | | | | | | | |
| NDVI at first two years of follow-up | | | | | | | | | |
|  | n=3,724, 803 events | |  | n=4,325, 1,689 events | | | n=7,970, 3,235 events | | |
|  | 0.90  (0.82, 1.00) | 1.00  (0.90, 1.11) | 0.99  (0.88, 1.12) | 1.00  (0.94, 1.06) | 0.98  (0.92, 1.05) | 0.93  (0.86, 1.02) | 0.91  (0.87, 0.95) | 0.92  (0.88, 0.97) | 0.94  (0.89, 1.00) |
| *** P_for interaction_ = 0.09 | | | | | | | | | |

Measured in a continuous scale, greenness scaled to a 1–IQR increment (0.047). Sample size and the number of events are reported for the full analytic dataset. ^a^ Adjusted for sex (age was accounted for in the time scale); ^b^ additionally adjusted for smoking, preexisting comorbidities (diabetes, hypertension and stroke), neighborhood SES and year of study entry; ^c^ additionally adjusted for living in an urban locality, exposure to traffic-related air pollution, and walkability index measured within a buffer of 800m around residential location [excluding 266 (6.7%) free of CHD, 828 (17.4%) stable and 1,403 (16.1%) acute participants due to missing information on these variables). P for interaction is derived from a Cox model including CHD status and continuous NDVI as main effects and an interaction term for CHD*NDVI in the pooled cohort. CHD, coronary heart disease; CI, confidence interval; HR, hazard ratio; IQR, interquartile range; NDVI, normalized difference vegetation index.

**Table S10. Association between greenness exposure measured at alternative spatial resolutions (indicated by Normalized Difference Vegetation Index in 100 m and 300 m around residential location) and all-cause mortality by CHD status (n=17,431)**

|  | Free of CHD  (n=3,959, 820 events) | | | | | | Stable CHD  (n=4,771, 1,824 events) | | | | | | | Acute CHD  (n=8,701, 3,411 events) | | | | |
| --- | --- | --- | --- | --- | --- | --- | --- | --- | --- | --- | --- | --- | --- | --- | --- | --- | --- | --- |
|  | **Model 1^a^** | | **Model 2^b^** | | **Model 3^c^** | | **Model 1^a^** | | **Model 2^b^** | | **Model 3^c^** | | | **Model 1^a^** | | **Model 2^b^** | **Model 3^c^** | |
| 100 m buffer | | | | | | | | | | | | | | | | | | |
| Greenness modeled on a continuous scale | | | | | | | | | | | | | | | | | | |
| - | 0.89  (0.81, 0.98) | | 1.03  (0.93, 1.15) | | 1.04  (0.92, 1.18) | | 0.95  (0.90, 1.01) | | 0.97  (0.91, 1.03) | | 0.93  (0.85, 1.02) | | | 0.85  (0.81, 0.88) | | 0.88  (0.84, 0.93) | 0.91  (0.86, 0.97) | |
| Categorical classification of greenness exposure | | | | | | | | | | | | | | | | | | |
| Low | **1** | | **1** | | **1** | | **1** | | **1** | | **1** | | | **1** | | **1** | **1** | |
| Medium | 0.85  (0.72, 1.00) | | 0.96  (0.81, 1.14) | | 0.95  (0.80, 1.13) | | 0.92  (0.82, 1.03) | | 0.94  (0.84, 1.05) | | 0.93  (0.83, 1.05) | | | 0.91  (0.84, 0.98) | | 0.96  (0.88, 1.04) | 1.00  (0.92, 1.09) | |
| High | 0.76  (0.64, 0.90) | | 0.96  (0.80, 1.16) | | 0.95  (0.77, 1.17) | | 0.94  (0.84, 1.05) | | 0.99  (0.88, 1.12) | | 0.98  (0.85, 1.13) | | | 0.77  (0.71, 0.84) | | 0.84  (0.77, 0.92) | 0.90  (0.81, 0.99) | |
| *** P_for interaction_ = 0.01 | | | | | | | | | | | | | | | | | | |
| 300 m buffer | | | | | | | | | | | | | | | | | | |
| Greenness modeled on a continuous scale | | | | | | | | | | | | | | | | | | |
| - | | 0.89  (0.80, 0.98) | | 1.03  (0.92, 1.14) | | 1.02  (0.90, 1.16) | | 0.98  (0.92, 1.03) | | 0.99  (0.92, 1.05) | | 0.93  (0.84, 1.02) | 0.83  (0.79, 0.87) | | 0.87  (0.82, 0.91) | | | 0.89  (0.83, 0.95) |
| Categorical classification of greenness exposure | | | | | | | | | | | | | | | | | | |
| Low | | **1** | | **1** | | **1** | | **1** | | **1** | | **1** | **1** | | **1** | | | **1** |
| Medium | | 0.82  (0.70, 0.96) | | 0.96  (0.81, 1.13) | | 0.95  (0.80, 1.13) | | 0.89  (0.80, 1.00) | | 0.89  (0.79, 0.99) | | 0.86  (0.76, 0.97) | 0.90  (0.83, 0.98) | | 0.95  (0.87, 1.03) | | | 0.98  (0.90, 1.06) |
| High | | 0.78  (0.65, 0.92) | | 1.01  (0.84, 1.22) | | 1.02  (0.83, 1.25) | | 0.89  (0.80, 1.00) | | 0.92  (0.81, 1.03) | | 0.86  (0.74, 1.00) | 0.76  (0.70, 0.83) | | 0.82  (0.75, 0.90) | | | 0.87  (0.79, 0.97) |
| *** P_for interaction_ = 0.003 | | | | | | | | | | | | | | | | | | |

Measured in a continuous scale, greenness scaled to a 1–IQR increment (0.042 for both 100 m and 300 m NDVI). Sample size and number of events are reported for the full analytic dataset. ^a^ Adjusted for sex (age was accounted for in the time scale); ^b^ additionally adjusted for smoking, preexisting comorbidities (diabetes, hypertension and stroke), neighborhood SES and year of study entry; ^c^ additionally adjusted for living in an urban locality, exposure to traffic-related air pollution, and walkability index measured within a buffer of 400m around residential location [excluding 277 (7.0%) free of CHD, 640 (13.4%) stable and 1,005 (11.6%) acute participants due to missing information on these variables). P for interaction is derived from a Cox model including CHD status and continuous NDVI as main effects and an interaction term for CHD*NDVI exposure in the pooled cohort. CHD, coronary heart disease; CI, confidence interval; HR, hazard ratio; IQR, interquartile range; NDVI, normalized difference vegetation index.

**Table S11. Association between greenness exposure (indicated by Normalized Difference Vegetation Index in 800 m around residential location) and all-cause mortality using a refined acute CHD categorization**

|  | Free of CHD  (n=3,959, 820 events) | | | Stable CHD  (n=4,771, 1,824 events) | | | Unstable angina pectoris  (n=3,287, 1,183 events) | | | Acute myocardial infarction  (n=5,414, 2,228 events) | | |
| --- | --- | --- | --- | --- | --- | --- | --- | --- | --- | --- | --- | --- |
|  | **Model 1^a^** | **Model 2^b^** | **Model 3^c^** | **Model 1^a^** | **Model 2^b^** | **Model 3^c^** | **Model 1^a^** | **Model 2^b^** | **Model 3^c^** | **Model 1^a^** | **Model 2^b^** | **Model 3^c^** |
| Greenness modeled on a continuous scale | | | | | | | | | | | | |
| - | **0.92**  (0.83, 1.02) | **1.02**  (0.92, 1.14) | **1.01**  (0.88, 1.16) | **0.99**  (0.94, 1.05) | **0.99**  (0.92, 1.05) | **0.90**  (0.81, 0.99) | **0.87**  (0.81, 0.94) | **0.89**  (0.81, 0.97) | **0.92**  (0.82, 1.03) | **0.83**  (0.78, 0.88) | **0.86**  (0.81, 0.92) | **0.84**  (0.78, 0.92) |
| Categorical classification of greenness exposure | | | | | | | | | | | | |
| Low | **1** | **1** | **1** | **1** | **1** | **1** | **1** | **1** | **1** |  |  |  |
| Medium | 0.84  (0.71, 0.98) | 0.94  (0.80, 1.11) | 0.95  (0.79, 1.13) | 0.91  (0.81, 1.02) | 0.90  (0.80, 1.01) | 0.87  (0.77, 0.99) | 0.86  (0.75, 0.99) | 0.90  (0.78, 1.04) | 0.94  (0.81, 1.10) | 0.89  (0.80, 0.98) | 0.96  (0.87, 1.06) | 0.97  (0.88, 1.08) |
| High | 0.83  (0.70, 0.99) | 1.01  (0.84, 1.22) | 1.01  (0.81, 1.25) | 0.92  (0.82, 1.03) | 0.92  (0.82, 1.03) | 0.84  (0.72, 0.97) | 0.81  (0.70, 0.93) | 0.84  (0.73, 0.97) | 0.89  (0.75, 1.06) | 0.73  (0.66, 0.81) | 0.78  (0.70, 0.88) | 0.78  (0.68, 0.89) |
| *** P_for interaction_ < 0.001 | | | | | | | | | |  |  |  |

Measured in a continuous scale, greenness scaled to a 1–IQR width increment (0.047). Results for 'Free of CHD' and 'Stable CHD' are consistent with those presented in the main analysis and are provided here for a convenient comparison. ^a^ Adjusted for sex (age was accounted for in the time scale); ^b^ additionally adjusted for smoking, preexisting comorbidities (diabetes, hypertension and stroke), neighborhood SES and year of study entry; ^c^ additionally adjusted for living in an urban locality, exposure to traffic-related air pollution, and walkability index; this analysis included 2,856 patients with unstable angina pectoris and 4,840 MI patients (1,005 patients were removed due to missing traffic-related air pollution and walkability data). P for interaction is derived from a Cox model including CHD status and continuous NDVI as main effects and an interaction term for CHD*NDVI in the pooled cohort. CHD, coronary heart disease; CI, confidence interval; HR, hazard ratio; IQR, interquartile range; NDVI, normalized difference vegetation index, MI, myocardial infarction.

**References**

1. Sallis JF, Floyd MF, Rodríguez DA, Saelens BE. Role of built environments in physical activity, obesity, and cardiovascular disease. *Circulation* 2012; **125**(5): 729-37.

2. Handy SL, Boarnet MG, Ewing R, Killingsworth RE. How the built environment affects physical activity: views from urban planning. *American journal of preventive medicine* 2002; **23**(2 Suppl): 64-73.

3. James P, Kioumourtzoglou MA, Hart JE, Banay RF, Kloog I, Laden F. Interrelationships Between Walkability, Air Pollution, Greenness, and Body Mass Index. *Epidemiology (Cambridge, Mass)* 2017; **28**(6): 780-8.

4. India-Aldana S, Rundle AG, Zeleniuch-Jacquotte A, et al. Neighborhood Walkability and Mortality in a Prospective Cohort of Women. *Epidemiology (Cambridge, Mass)* 2021; **32**(6): 763-72.

5. Lang JJ, Pinault L, Colley RC, et al. Neighbourhood walkability and mortality: Findings from a 15-year follow-up of a nationally representative cohort of Canadian adults in urban areas. *Environment international* 2022; **161**: 107141.

6. Leslie E, Coffee N, Frank L, Owen N, Bauman A, Hugo G. Walkability of local communities: Using geographic information systems to objectively assess relevant environmental attributes. *Health & Place* 2007; **13**(1): 111-22.

7. Zagorskas J. GIS-Based Modelling and Estimation of Land Use Mix in Urban Environment. *International Journal of Education* 2016; **01**.

8. Frank LD, Sallis JF, Saelens BE, et al. The development of a walkability index: application to the Neighborhood Quality of Life Study. *British journal of sports medicine* 2010; **44**(13): 924-33.
